# Climate-catchment-soil control on hydrological droughts in peninsular India

**DOI:** 10.1038/s41598-022-11293-7

**Published:** 2022-05-15

**Authors:** Poulomi Ganguli, Bhupinderjeet Singh, Nagarjuna N. Reddy, Aparna Raut, Debasish Mishra, Bhabani Sankar Das

**Affiliations:** Agricultural and Food Engineering Department, Indian Institute of Technology Kharagpur, Kharagpur, West Bengal 721302 India

**Keywords:** Environmental sciences, Hydrology, Natural hazards, Civil engineering

## Abstract

Most land surface system models and observational assessments ignore detailed soil characteristics while describing the drought attributes such as growth, duration, recovery, and the termination rate of the event. With the national-scale digital soil maps available for India, we assessed the climate-catchment-soil nexus using daily observed streamflow records from 98 sites in tropical rain-dominated catchments of peninsular India (8–25° N, 72–86° E). Results indicated that climate-catchment-soil properties may control hydrological drought attributes to the tune of 14–70%. While terrain features are dominant drivers for drought growth, contributing around 50% variability, soil attributes contribute ~ 71.5% variability in drought duration. Finally, soil and climatic factors together control the resilience and termination rate. The most relevant climate characteristics are potential evapotranspiration, soil moisture, rainfall, and temperature; temperature and soil moisture are dominant controls for streamflow drought resilience. Among different soil properties, soil organic carbon (SOC) stock could resist drought propagation, despite low-carbon soils across the Indian subcontinent. The findings highlight the need for accounting feedback among climate, soil, and topographical properties in catchment-scale drought propagations.

## Introduction

Peninsular River Basins (PRB) of India (8–25° N, 72–86° E) are facing increasingly severe droughts and water scarcity^1–3^. Climate change and an ever-growing population further strain locally-available surface water^4^ gradually push the region towards a ‘day-zero’ situation^5^. Krishna and Godavari are the two major rivers in PRB and both are rain-fed. Failures and delays in southwest (June to September) or northeastern (October–December) monsoon^6–8^ in this region trigger below-normal streamflow and hydrological droughts^9^ in varying intensities. Even with decades of catchment-scale drought propagation studies^2,8,11,12^, it is not clear how a given river basin develops into a “drought-rich” or “drought-poor” region. Climate and catchment control on hydrological droughts are more or less known^13–17^; however, no studies have attempted to examine how varying soil conditions influence these controls. With the availability of a national-scale digital soil map^18^, here we explore the climate-catchment-soil control on hydrological droughts and identify key drought drivers (KDD) for drought propagation.

The observation-based drought assessments in India either focused on standardized precipitation or evapotranspiration indices that compare departures of the variables from its long-term averages or assumed a simple linear relationship between precipitation and potential evapotranspiration, e.g., aridity index^2,19–24^. Based on a semi-distributed hydrological model, Variable Infiltration Capacity (VIC), a few studies^10,11^ have investigated the propagation of meteorological to hydrological droughts at several catchments of India, considering multiple temporal scales using the standardized monthly anomalies of streamflow and precipitation-based indices. However, these monthly anomaly-based indices often rely on fixed percentile thresholds for defining drought episodes. While most decision-makers and stakeholders emphasize the use of particular discharge levels rather than anomalies as triggers for management actions^25^, places where pronounced seasonality dominates, the variable thresholds capture the seasonal variations better by following the seasonal amplitude and prevents the natural low flow season considered under drought^15,26^.

At a large scale, a few studies have explored the spatiotemporal pattern of drought recovery over North America^27,28^ and across the Globe^29–31^. However, at a global-scale, two of the studies^29,30^ presents diametrically opposite insights regarding the timing of drought recovery in the tropics and the high latitude continents. In particular, Yu et al.^30^ reported shortest drought recovery time in the tropics and high latitudes (< 4 months), whereas Schwalm et al.^29^ reported longer than 12 months drought recovery time for these regions. A subsequent study by Liu et al.^31^ reconciled that contradictory findings in global drought assessments are the artifacts of different approaches adopted in drought identification methods and recovery level definitions. Further, all three assessments^29–31^ considered a monthly temporal resolution of underlying drivers influencing drought mechanism, which may fail to identify small drought development phases. Typically a higher temporal resolution is required for predictive modelling of extreme events in both space and time, which helps in issuing timely alerts related to regional water scarcity^32,33^. Although few studies^27,28,34–37^ have assessed droughts using low flows and streamflow droughts using streamflow records of daily temporal resolution, their analyses domains are constrained to either mid and high latitude continents, where the nature of streamflow often follow the nival, pluvial and mixed flow regimes. Insights from these studies may not be generalizable for other climatic regions, especially in tropics where marked seasonality is apparent in streamflow records^38^.

**Figure 1:**
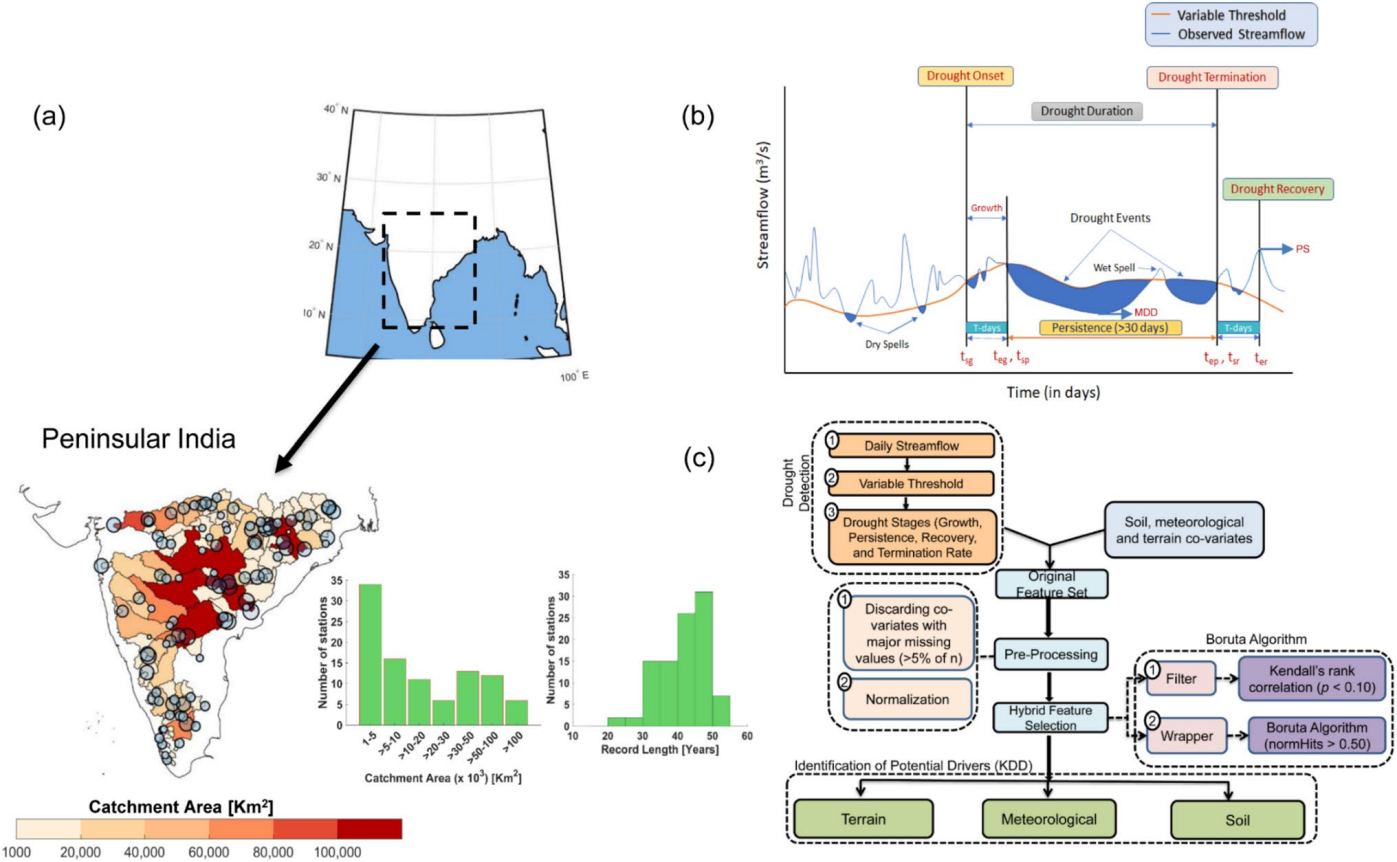
Distribution of Stream gauges, Drought Characteristics and Conceptual Diagram Illustrating Key Drought Drivers (KDD) Detection. **(a)** Location of stream gauges within each catchment. The size of bubbles shows the record length, which is proportional to the sample size (in years). Histograms show the distribution of catchment area (in km^2^), and available record lengths (in years). (**b)** Identification of drought characteristics using daily variable threshold approach. The blue shaded region depicts streamflow deficit. The *t*_*sg*_ and *t*_*eg*_ represent the start and end of the growth period. Likewise, *t*_*sp*_ and *t*_*ep*_ indicate the initiation and termination of the drought persistence stage. *t*_*sr*_ and *t*_*er*_ denote the initiation and termination of the drought recovery, MDD and PS indicate maximum drought deficit volume during the persistence stage and peak surplus flow after drought termination. (**c**) Detection of KDD using random forest-based feature selection algorithm. The threshold criterion, normHits > 0.50 indicates only those features are selected that show higher 'importance' than their shadow attributes (obtained by random permutation of features) for more than 50% of total iterations. The figures are prepared in MATLAB R2020b (academic version), MS Office Power point 2016 and then organized in Adobe Photoshop CS3 Desktop (http://www.adobe.com) [Software].

Considering these research gaps, we examine the following research questions: (i) Is there any systematic method to understand drought development and its propagation phases for tropical pluvial river basins? (ii) Are there any spatial and/or temporal clustering behavior of streamflow droughts are apparent over PRB, and what would be the nature of drought characteristics over these identified regimes? (iii) How do climate-catchment and soil properties interact to control catchment-scale drought phases and its characteristics over PRB? These insights add value in drought preparedness and shaping policy recommendations for agricultural and industrial sectors^39,40^. We used daily observed streamflow records of past 50 years (1965–2019) from 98 stream gauges over PRB in a multi-stage framework^37,41^ (Fig. 1) to quantify the contiguity in locations and time of occurrence of hydrologic droughts (the space–time clustering^42^ or synchronicity in drought properties) and identify potential KDDs from a wide range of climate, soil, and terrain attributes (Fig. 1, Supplementary Fig. S1, Table S1). We applied a daily variable threshold approach to derive streamflow droughts by developing 366 (additional for leap year) flow duration curves using continuous streamflow records^26^ (“Methods”). While we obtain meteorological and catchment-specific geospatial attributes from the archived database^43–47^, the soil attributes are derived from a recently developed digital soil database of India^18^ (see Data and method section). We show the extent to which climate, catchment and soil attributes influences and co-vary with catchment-scale drought characteristics (“Methods”), such as growth, persistence (duration and frequency or number of events), recovery, and drought termination rate (DTR). Specifically, we investigate how soil organic carbon (SOC) influence the growth, persistence, and recovery of droughts over PRB given that the Indian soils are typically low in SOC contents^18,48^.

## Space–time synchronicity in drought responses

Previous studies^10–13,29^ have used gridded hydrometeorological forcing with a coarser temporal resolution to identify drought clusters over PRB. Here, we identify the temporal evolution of drought characteristics using continuous daily streamflow records, namely, drought growth, persistence, recovery and the DTR (See “Methods”; Fig. 1b,c). Then, we identify drought regimes by applying a clustering algorithm to 98 gauges across PRB based on nine catchment-scale drought attributes (see “Methods”): (i) latitude and longitude of stream gauges; (ii) drought properties, *i.e.,* mean and the maximum drought duration, and mean and the maximum drought deficit volume; (iii) catchment properties, such as the baseflow index (BFI; see “Methods”)^49^ and catchment area, and (iv) seasonality^50^ in drought termination. We show the temporal evolution of drought characteristics and identify the presence of “drought rich” and “drought poor” periods over the past five decades using the Hovmöller diagram (Fig. S2). The decadal pattern of events (during the time-window 1979–80, 1989–90, 2001–02, 2008–10) shows over 30% of the areas are drought-affected. Further, we identify spatial clustering of persistent droughts over several regions, primarily concentrated between latitudinal belts 13° and 20° N between 2001 to 2005, including two major historical hydrological drought events spanning the periods, 2000–2001 and 2003–2004 (ref.^10^). The droughts over India are typically associated with prolonged dry spells due to abnormally low and erratic monsoon rainfall that can last over a season or longer, extending over a large spatial scale^7,51–53^. Although based on instrumental all India rainfall observations from 1901 to 2010, Pai et al.^54^ reported over twenty drought years with severity varying from moderate to intense: the iconic droughts of July 2002 led to below average rainfall and droughts in large part of the western peninsula^55^. Interestingly, rainfall during the monsoon season over India is linked with “teleconnections” through which sea surface temperature (SST) induces large-scale atmospheric patterns that trigger the development of dry conditions and major monsoon failures^56^. Stronger El Niño droughts over the Indian peninsula are due to decreased east-ward moisture flux over the Arabian Sea during the warm El Niño episodes. In contrast, during non-ENSO (El Niño Southern Oscillation) years, the emergence of extreme wet/dry spells is due to anomalous moisture convergence driven by surface pressure gradients surrounding the peninsular region^57^. A co-occurring Indian Ocean Dipole (IOD) mode further modulates El Niño Southern Oscillation (ENSO), which influences monsoon season droughts^58^: while positive phase of IOD is conducive to wetter-than-normal conditions, a co-occurring positive (negative) IOD significantly reduces the impact of the El Niño (La Niña) on the Indian monsoon. While a weakening of ENSO versus summer monsoon rainfall was apparent since the 1970s, a restoration of this relationship since 1999/2000 is due to the inter-decadal transition of ENSO evolution and the SST over the tropical Atlantic^59^.

**Figure 2:**
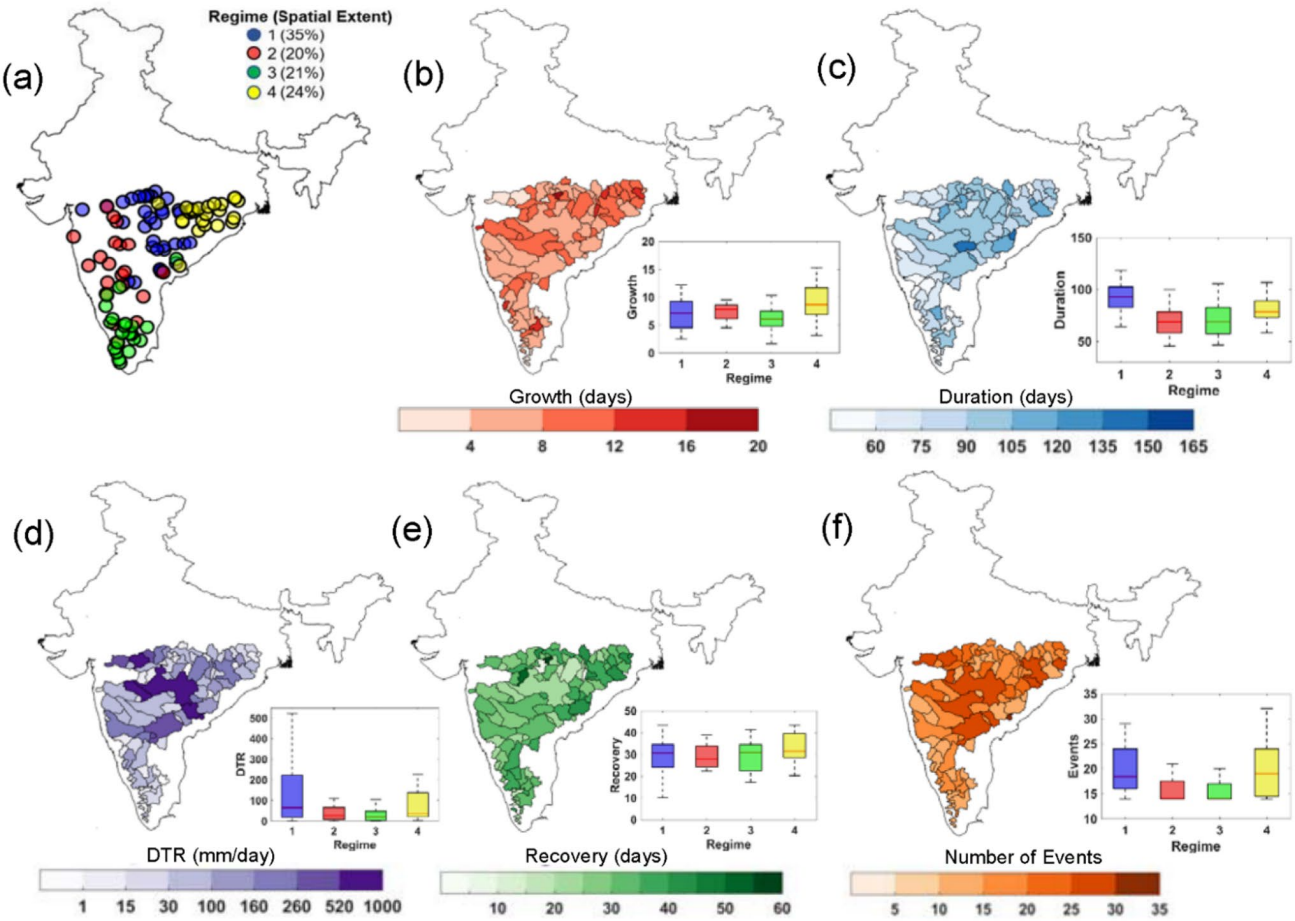
Identification of Drought Regimes and Illustration of Catchment-scale Drought Properties. **(a)** Regionalization of droughts based on drought characteristics using fuzzy c means clustering algorithm (see “Methods”); *n* indicates the number of sites detected within each cluster. **(b–f)** Spatial distributions of drought characteristics during 1965–2018 time window: **(b)** drought growth (in days) **(c)** duration (days) **(d)** drought termination rate or DTR (mm/day) (**e**) recovery period (in days) **(f)** drought frequency or number of events. The boxplots in inset show the variability in drought properties among the identified clusters. Box center marks (red lines) are medians; box bottom and top edges show 25th and 75th percentiles respectively, whereas the spread of the boxes indicates interquartile range; whiskers indicate q75 + 1.5(q75 − q25) and q25 − 1.5(q75 − q25), where q is the quantiles of variables. The shades of boxes in purple, red, green and yellow indicate streamflow drought regimes 1–4, based on selected drought attributes. The figures are prepared in MATLAB R2020b (academic version) and organized in MS Office Power point 2016 [Software].

To explore the nature of hydrological drought responses on a regional scale, we delineate the collection of sites based on fuzzy c-means clustering^60,61^ (see “Methods” and the Supplementary Information SI 1.2). A study by Ahmadi et al.^37^ showed characterizing droughts into different stages or properties provide better understanding of temporal and spatial coherence of localized drought events. Further, Yaeger et al.^62^ showed that only accounting geomorphological features and drought attributes may not provide a credible estimates of the homogenous region. Hence, we introduce the seasonality of drought termination month, represented by the mean date of drought termination, to identify homogenous regions (see “Methods”). The regionalization of hydrological droughts based on drought properties involves the Principal Component Analysis (PCA) followed by fuzzy c-means clustering method^63^ (See SI 1.2). Based on PCA and fuzzy clustering, we identify the optimal number of drought regimes (i.e., represented by a cluster of sites based on drought-specific attributes) as four (Fig. 2a). We find that collectively the first six principal components (PCs) explain the ≈ 94% variability of the streamflow droughts characteristics (Fig. S3a); therefore, only the first six PCs are used for identifying drought clusters. The biplot of the top two PCs of the selected attributes shows (Fig. S3b) that the maximum and mean drought durations have notable contribution to the first PC. On the other hand, for the second PC, the seasonality of drought termination, showed the significant contribution. The mean deficit volume and the catchment area did not significantly contribute to the first two PCs. The BFI showed a negative correlation with both these PCs. Geospatial locations and drought durations significantly contributed to the spatial variations in clusters 1 and 4 and the mean termination date contributed to the spatial variations in cluster 2. Finally, the BFI that inherently embeds the effect of geology and soil permeability is the major contributor to variations in cluster 3.

Figure 2a shows the delineated hydrological drought regimes, a large fraction of stream gauges located across the central part of PRB is under regime 1 with 35% spatial extent; whereas regimes 2–4 contain 20–24% of gauges. Most of the clusters are disjoint with only a few minor overlaps among cluster members indicates the detected regimes are optimal in numbers and credible enough to show observed drought characteristics at a regional level. Among the obtained drought clusters, regime 1 lies in the core monsoon region; regime 2 is located across arid and semi-arid part of the peninsula; whereas regimes 3 and 4 are influenced by the northeast monsoon during October-December^10^. It should be noted that the areal extents of regimes 3 and 4 are close to the clusters ‘NEI’ and ‘SI’ in ref.^10^, where authors have used precipitation and model-simulated ‘Integrated Drought Index’^10^ at a monthly temporal resolution as a forcing variables for the clustering algorithm. Figure 2b–f shows the spatial distribution of drought characteristics during 1965–2018 time window. Most catchments located in Central (i.e., catchments of Godavari, and Narmada) and a few of eastern (Subarnarekha and Mahanadi) river basins (Fig. S1) reported a large growth period, often more than a week (Fig. 2b) with frequent drought (Fig. 2f) events. The average drought duration in the catchments of Godavari and Narmada from regimes 1 and 2 ranges more than 50 to 100 days. In particular, the catchments in regime 1 show a large variation in DTR often exceeding 250 mm/day (Fig. 2d) with a recovery length more than a month (Fig. 2e). The spatial distribution of seasonality in drought termination (Fig. S4) shows high regularity in drought termination for regimes 1 and 2 with average seasonality of more than 0.5. The catchments in regime 1, which includes 74% sub-basins from Narmada, and the Godavari in Central India and remaining from Krishna, and Mahanadi basins contains large watershed area and show persistently longer drought episodes with average termination period during mid-monsoon season during the month of September. Temporal evolution of drought characteristics during 2000–2005 time window for rivers in Central India (regime 1) shows (Fig. S5) the growth of droughts initiated during the month of August in 2000, which lasted until early 2001; subsequently, the majority of stations showed recovery in the monsoon season of the same year (i.e., in June 2001). During the year 2003–2005, we note the presence of multi-season persistent droughts, especially towards the South of 20° N, which lasted for more than a year (from March 2004 to July 2005) in this region. The rivers in this region contains low BFI with a median value around 0.3. Further, this region often accompanied by strong local heating of the black soils with high PET^64^, which could lead to low baseflow yield in this region^65^. The low BFI, indicates rivers in this regime is associated with small catchment memory due to less permeable soil layers that force rainfall to flow quickly to the stream. Due to low permeability of soil, the region may experience more minor drought events that have short duration.

The sub-basins in Regime 2 shows relatively fewer drought events than other regions with relatively low average drought duration (less than 100 days; Fig. 2c) and is associated with the lowest average recovery period (average recovery less than a month; Fig. 2e). This regime includes 70% of sub-basins from Krishna, Tapi, and the Godavari River basins (Fig. S1) with moderately large catchments areas. The most severe drought that occurred in Regime 2 lasted around 250 days (August 2003 to April 2004; Fig. S5). For gauges located in this regime, the drought terminations ranges between August and December months with median termination during post-monsoon season in October (Fig. S4). The values of BFI tend to be the lowest for this regime as compared to others, with a median BFI value of 0.25 (Fig. S4). Interestingly, the rivers in this regime shows a strong seasonality in the mean timing of drought termination with the strength of seasonality close to 0.8, indicating high persistence in drought termination, i.e., all streamflow droughts at a particular site occur on the same day of the years during the analysis time window^66^.

Regime 3, comprising nearly 60% of sub-basins from Cauvery and Krishna, and the rest from the southern peninsula region (e.g., Pampa, Periyar, Vaigai), experience the lowest number of droughts - on an average, 15–20 events (Fig. 2f), followed by a minimum variation in the DTR of < 15 mm/day (Fig. 2d). In general, the drought termination pattern in regime 3 does not show any specific trend with termination period scattered throughout the year with a large variation in seasonality strength; however, August is detected as the median termination month (Fig. S4). The rivers in this regime shows the highest BFI (with BFI > 0.5). The review of the literature^67,68^ shows that areas with lakes and reservoirs (e.g., Krishnaraj Sagara reservoir over Cauvery River in this region) have high values of BFI since outflows from surface water, such as lakes, reservoirs, and wetlands, can comprise a major portion of baseflow. The catchments with high BFI sustain the recharge and groundwater storage^65^, which results in large variation in drought termination months (or low seasonality in drought termination; Fig. S4).The analysis of 2000–05 time window for regime 3 shows (Fig. S5) the “drought-rich” periods exist after 2002, which persists between 2003 and 2005. By early 2003, the catchments of Cauvery and a few catchments in southern India (e.g., Pampa and Ponnaiyar) were also affected and remained under drought throughout the year, which recovered later in April–May 2004.

Finally, regime 4, comprising a majority of catchments across eastern peninsular India (87% of sub-basins from Mahanadi, Subarnarekha, and Brahmani and the rest from Baitarni and Godavari; Fig. S1) reported an average drought duration of more than two months with a large variability in drought frequency (15–30 events) (Fig. 2f). The average drought recovery length in this regime is relatively larger (Fig. 2e) and a large number of sites show a recovery period of more than 40 days. The most severe drought in regime 4 occurred in August 1979, which lasted until July 1980 (Fig. S2) and was considered as a severe drought in the literature^69,70^. The average drought termination period in this regime is mainly during post-monsoon period in November (Fig. S4) with termination months varies from October to December. The catchments in this regime showed the least regularity in drought termination (Fig. S4).

Overall, our analyses reveal the following: (*i*) majority of regimes (1, 2, and 4) show the average termination either in the monsoon (June–September) or post-monsoon (October-December) months, suggesting profound roles of southwest and northeast monsoon rainfalls in the termination of droughts. On the other hand, regime 3 showed no specific trend in drought termination seasonality with termination periods scattered throughout the year. (*ii*) Large spatial heterogeneity in drought responses indicates drought stages differ significantly across space and time, which could be a consequence of several factors including topography and morphological attributes of catchments, soil, and climatic controls^15,16,35^.

## Hot and cold spots of streamflow droughts

**Figure 3:**
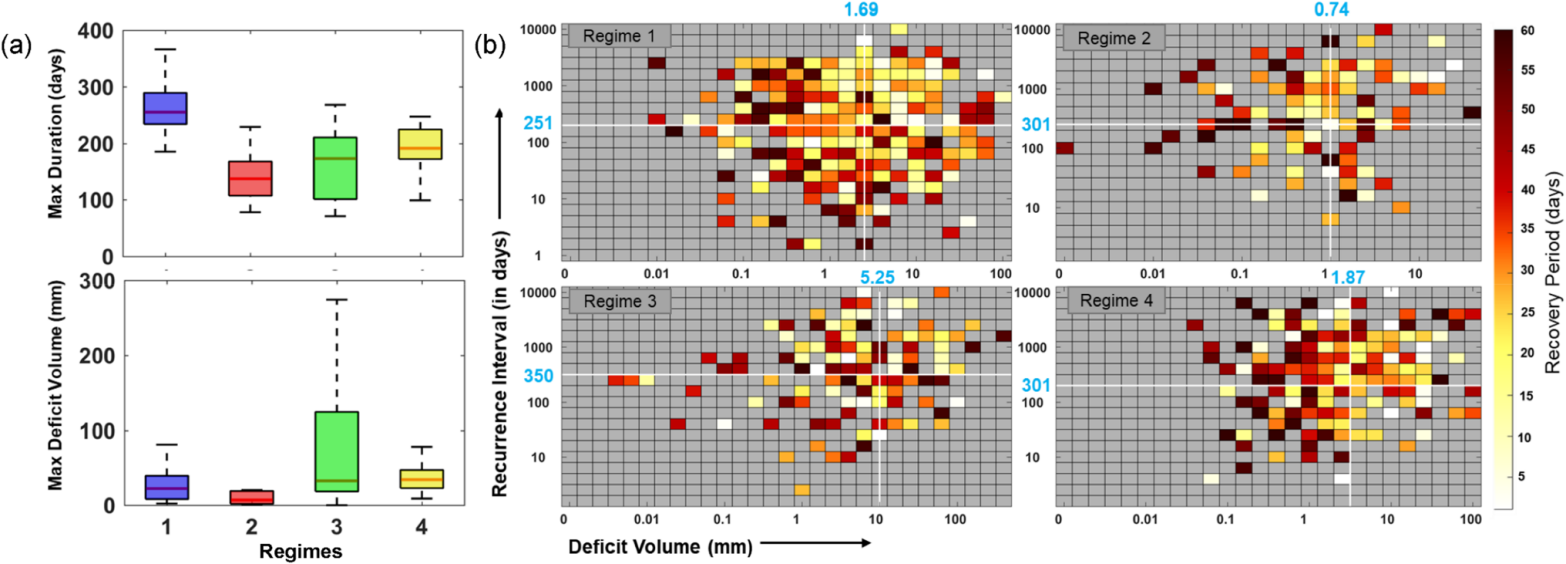
Variations in Drought Properties, the Maximum Deficit Volume , Maximum Duration, and Recovery Times among the Detected Clusters**. (a)** The boxplots showing interquartile range of selected drought attributes, the (maximum) duration and the deficit volume. **(b) **The recovery period as a function of deficit volume and recurrence interval (i.e., the time interval between two successive droughts but neglecting the first drought event) for the identified regimes. The shades of each pixel show the drought recovery period. The cells in grey indicates no observation. The straight lines in white perpendicular to the axes show the median deficit volume and the median recurrence interval for each region. The figures are prepared in MATLAB R2020b (academic version) and then organized in MS Office Power point 2016 [Software].

To further explain the nature of synchronicity in drought responses and identify vulnerable regions, we compare the maximum deficit volume and maximum duration of streamflow droughts (Fig. 3a). In addition, we present heat maps of drought deficit volume-recurrence interval-vs-recovery duration for different regimes (Fig. 3b). A large fraction of catchments in Regime 1 is characterized by moderately severe drought with a spatial average value of 1.7 mm; however, they experience long and persistent drought episodes  of more than 250 days (Fig. 3a). The rivers in this regime show an extended drought recovery period coinciding with a short return time or recurrence interval (within the range of 250 days; Fig. 3b).

On the other hand, regime 2 shows droughts with relatively longer recurrence interval accompanied by more than a month of recovery period. Droughts in this regime have the lowest deficit volume, with an average deficit volume of ~ 0.74 mm (Fig. 3a,b). This could be because catchments in this region show the lowest BFI values than others (Fig. S4), suggesting a minimum contribution towards groundwater recharge owing to relatively impermeable geology^16^. Regime 3 shows the largest average recovery length (Fig. 2e) with considerable variability in deficit volume—a few outlying events even led to deficit volume of more than 200 mm (See whisker length of the box plot in Fig. 3a). This region also shows considerable variability in drought seasonality (Fig. S4). Interestingly, more than 50% of sites show a recovery period of less than a month (shades of the pixels in Fig. 3b) with an average recurrence interval of 350 days (Fig. 3b), which is the largest among all regimes. A relatively small recovery period compounded by a large recurrence interval could be due to the largest baseflow indices of catchments in this region (Fig. S4), which indicate relatively permeable geology with substantial groundwater recharge.

Finally, regime 4 shows a contrasting pattern to regime 1, where droughts with relatively less deficit volume (i.e., < 1 mm) are coincided with a recovery period of more than a month. Further, a rare event characterized by a high deficit volume of more than 10 mm and a prolonged recurrence interval of more than 100 days often witnesses a low recovery period (typically less than a month; Fig. 3b, *bottom right corner*). A relatively long recovery period could be because of low baseflow indices for gauges in this region with a median value of less than 0.5 (Fig. S4), indicating rivers in this regime show greater peak flows with short lag time and lesser base flows^72,74^.

Overall, our analysis shows the following: (*i*) catchments in central peninsular India (13–23° N and 73–84° E) are exposed to frequent droughts compounded by a long recovery period, making it one of the most vulnerable regions where a chronic state may be reached when an incomplete recovery would coincide with another severe drought episode leading to an adverse consequence to land-carbon sink. Interestingly, this region contains relatively low SOC contents, as seen in the newly developed national SOC map^18^.

In contrast, (*ii*) catchments in regime 2 are characterized by relatively less severe droughts with a larger recovery period despite having the lowest BFI in regime 2. We hypothesize that streamflow drought resiliency in regime 2 could be partially linked to the high SOC content of the soil in the Western Ghat area of the PRB^18^—a high SOC may lead to an increase in soil water storage capacity resulting in a slowdown in severe drought occurrences. To investigate further, we compared the regional distributions of soil organic content (SOC; in %), available water content (AWC; in %) and annual average rainfall (Fig. S6). We show that despite regime 2 receives low annual average rainfall as compared to all regions, the relatively high surface (at 30 cm depth) and sub-surface (at 1 m depth) SOCs results in higher soil water storage potentials as manifested by the highest median AWC at sub-surface level among all regions. On the other hand, the low BFI at region 2 could be associated with climate, soil and geomorphologic properties. While soil controls the infiltration of water, the underlying aquifer properties control the storage and release of water to streams. Recently, Naveena et al.^64^ have detected emergence of a “hot blob” during the pre-monsoon season (end of March–May) over the south-central parts of the PRB, which promotes the accumulation of high temperature in this region. High clay content of black soils (region 2) further abets the sustenance of the “hot blob” resulting in higher frequencies of hot days, which could lead to low baseflow yields in this region^65^.

## Key drought drivers (KDD’s) influencing drought vulnerability

To provide a causal attribution of drought responses, we investigate the influence of several covariates, such as meteorological variables, soil properties, and catchment-specific terrain attributes (Table S1), totaling 89 hydrometeorological and morphological features. The Shapiro–Wilk test of drought variables as well as the covariates reveal that 85% of variables (i.e., 79 out of 93) show a strong deviation from normality assumption at a 10% significance level. The skewness and kurtosis values of covariates further confirm that the covariates exhibit a strong asymmetry (Fig. S7). The nonparametric dependence analysis (Kendall’s τ test; Fig. S8) suggests that the drought growth strongly depends on (significant positive dependence) terrain features in regime 1, from which topographic wetness index (TWI; see “Methods”) shows the highest correlation value of Kendall’s τ  = 0.39. This could be because the TWI^75,76^, which is a function of the local slope with the upslope contributing area per contour length, will be more likely in wet and relatively shallow soils with moderate slopes, where soil permeability increases with saturation. On the other hand, drought duration and recovery show (significant) negative dependence on SOC and stock (Kendall’s τ  < − 0.21). This may be due to moderately low SOC content in this region^18,48^.

In regime 2, the drought growth shows positive dependence to both soil and meteorological attributes, such as the mean temperature of April-July (Kendall’s τ  > 0.48) followed by pH and cation exchange capacity (CEC) values at 0.3 and 1 m soil depths (Kendall’s τ  > 0.47), respectively, whereas a negative dependence was observed for SOC content and SOC stock (Kendall’s τ  < − 0.35). In contrast, the recovery stage in this region shows more dependence on terrain features. In regime 3, the growth shows a strong positive dependence on different soil moisture covariates (Fig. S8). Further, there is high variability among factors influencing drought duration and recovery—in general, sub-basins show a strong negative dependence on soil organic content (Kendall’s τ  < − 0.44). In contrast, DTR fails to show any conclusive evidence of significantly strong dependence on any of the covariates. Finally, in regime 4, recovery and DTR show a moderately strong dependence with meteorological and terrain features, which is in the order of ± 0.4 (i.e., terrain feature slope show a significant negative dependence with drought recovery, Kendall’s τ_recovery_ = − 0.4 and a significant positive correlation with DTR, Kendall’s τ_DTR_ = 0.38).

Our analyses reveal a large proportion of gauges in regimes 2 and 3 that show a strong dependence on covariates. For example, in regime 2, 51% of catchments show strong dependence with covariates during growth phases. Likewise, drought persistency in regime 3 is largely controlled by 65% of covariates. Further, the drought resilience or recovery phase in regime 3 is more strongly influenced by terrain features as reflected by the largest BFI values followed by meteorological attributes. On the other hand, in regime 2 recovery phase shows a strong positive correlation, associated with terrain features. As noted earlier, the sub-basins in regime 2 show the lowest BFI, indicating a minimum baseflow contribution or groundwater replenishment, which results in a relatively long recovery period in this region. Our results corroborate with an earlier studies^73,74,77^, which showed low flows are often controlled by the soil and geology of the catchment.

**Figure 4:**
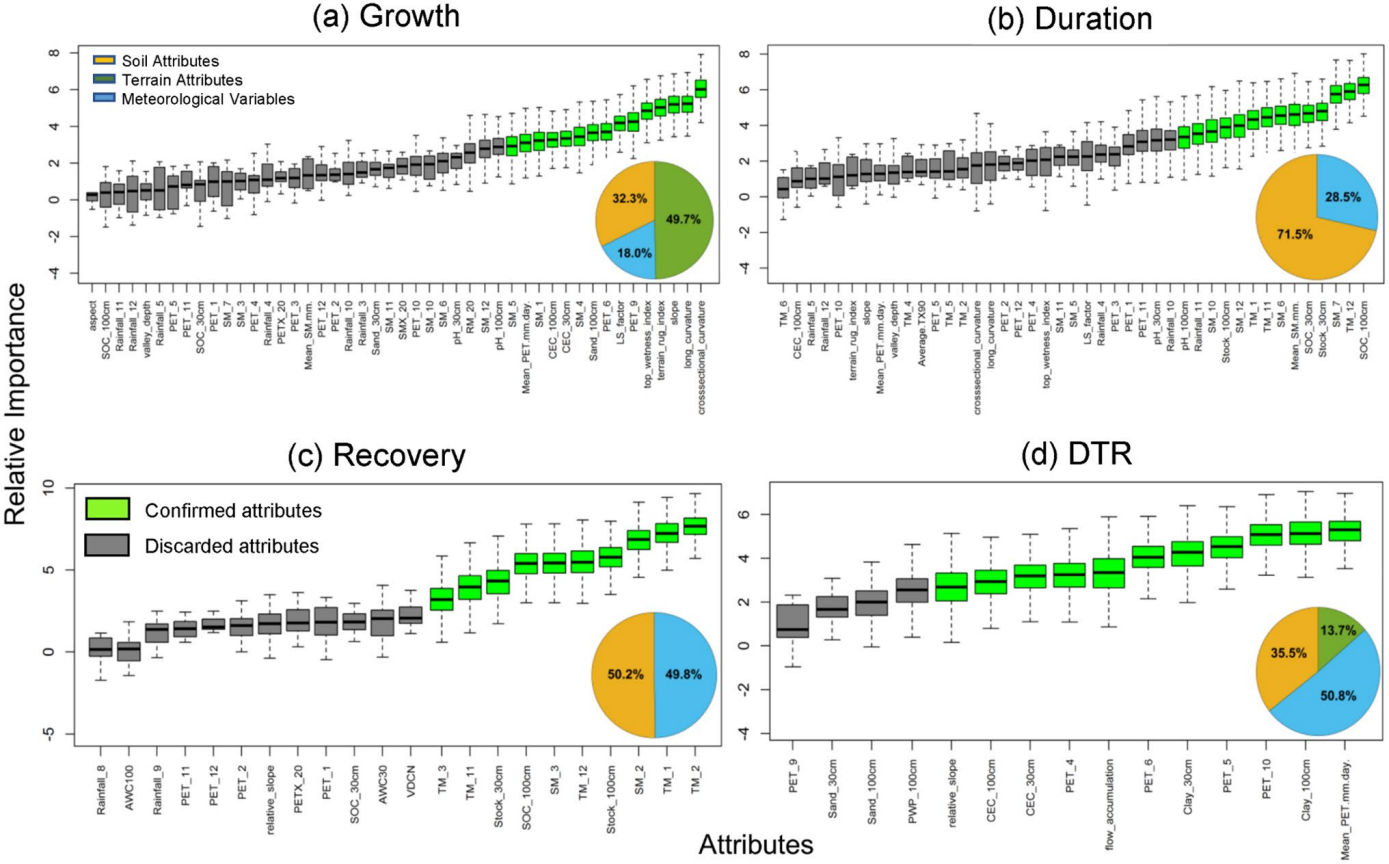
Potential Key Drought Drivers. The relative importance of key drought drivers is shown using box plots for various drought characteristics. The pie charts at the lower bottom corner show relative contribution of soil, terrain and meteorological variables in influencing drought stages. The x-axes show the soil-climate and topographical attributes; details of each of these attributes are described in Table S1. The legends applies to all figure panels. The figures are prepared in R-4.0.5 (64 bit) windows version and then organized in MS Office Power point 2016 [Software].

We employed a hybrid feature selection procedure consisting of filtering and wrapping through Boruta algorithm^78^ (see “Methods”) using all 89 covariates. The average sand contents at 1 m depth in the western part of the peninsula is relatively low as compared to the eastern and southern part of the peninsula, which influences the drought growth for gauges in this region (Fig. S9a), whereas a relatively high clay content in this region affects average drought termination rate (Fig. S9d). The SOC content and SOC stock at 1 m depth over a large portion of the landmass is consistently low (Fig. S9b-c). Among three KDD categories (soil, hydro-meteorological and terrain), drought growth appears to be most influenced by ~ 17% (15 out of 89) attributes (see Fig. 4a), e.g., the cross-sectional (Kendall’s τ  = − 0.23) and longitudinal (Kendall’s τ  = 0.22) curvatures, slope (Kendall’s τ  = − 0.23), and terrain roughness index (Kendall’s τ  = − 0.23) in addition to sand content (Kendall’s τ  =—0.14), CEC (Kendall’s τ  = 0.20), and soil moisture for the months of January (Kendall’s τ  = − 0.18), April (Kendall’s τ  = − 0.19), and May (Kendall’s τ  = − 0.21), denoting the influence of soil moisture on drought growth in the transition months from winter to spring and spring to summer. Drought growth shows a strong dependence on hydro-meteorological factors, such as average potential evapotranspiration (PET) at the onset (Kendall’s τ  = 0.14 for June) and retrieval (Kendall’s τ  = 0.17 for September) months of monsoon. This could be because of feedback between soil moisture and surface water availability (precipitation minus evapotranspiration, *P-E*). In water-limited regions, the soil moisture is shown to modulate evapotranspiration, which positively feedbacks precipitation via moisture recycling^79,80^. The drought duration showed strong dependence on soil properties, primarily SOC and SOC stock and mean monthly winter (November–December) soil moisture and temperature regimes. However, no terrain features are found to be critical in influencing drought duration. In general, soils with low SOC contents and moisture deficits during post-monsoon seasons will have a longer drought duration. Likewise, drought recovery appears to be largely dependent on mean monthly soil moisture contents during February and March (Kendall’s τ  = 0.12), mean temperature of February (Kendall’s τ  = − 0.17) and January (Kendall’s τ  = − 0.18), SOC contents, and SOC stocks of top 1 m soil profile (Kendall’s τ  = − 0.21). This agrees qualitatively with findings from an earlier study^81^, which showed that temperature strongly influences streamflow-based drought characteristics such as spatial extent and duration. Further, SOC controls the soil moisture levels and, in turn, drought development and termination stages (Fig. S9)^48,82^.

Interestingly, Fig. 4b confirms that the early monsoon (June-July) soil moisture conditions and winter (primarily between November and December) temperature notably impact on drought duration. On the other hand, drought recovery heavily depends on the soil moisture regime during the spring (February–March) and the temperature conditions during the winter (November–January) until the end of the spring (March-end) season. Likewise, the DTR is typically influenced by only 12% (11 out of 89) attributes (Fig. 4d). An apparent positive dependence between PET (Kendall’s τ  = 0.17), clay content (Kendall’s τ  = 0.16), and CEC (Kendall’s τ  = 0.14) with DTR suggests the inherent ability of soils coupled with hydro-meteorological factors to accelerate or cease prevailing desiccation. These are further aided by terrain factors such as flow accumulation (Kendall’s τ  = 0.22) and relative slope (Kendall’s τ  = 0.18) in governing the rate of drought termination. Overall, our results show that drought growth is largely controlled by terrain attributes ~ 50% of total covariates; drought persistency is mostly controlled by soil attributes accounting for more than 70% of all three covariates. Interestingly, drought recovery is equally controlled by hydroclimatic and soil properties with little or no role of terrain attributes, whereas DTR is primarily controlled by hydroclimatic (~ 51% share) and soil (~ 35% share) factors together.

Our analyses suggest the following: (*i*) Considering peninsular catchments as a whole, terrain features largely control drought growth; soil attributes contribute more than 70% in drought persistency; whereas DTR is largely controlled by meteorological attributes. In addition, drought resiliency is equally impacted by soil and meteorological attributes. (*ii*) Considering homogeneous drought regimes, a large proportion of soil-meteorological and catchment properties in regimes 2 and 3 show a strong dependence on growth (for regime 2) and persistence (for regime 3) phases, respectively. Further, drought recovery in regime 3 shows a strong anticorrelation with soil and terrain features, whereas a strong positive dependence on meteorological attributes, primarily with PET. The relatively small recovery period (often less than a month) of most of the  catchments compounded by a large recurrence interval at regime 3 could be attributed to the largest baseflow yields of catchments, which is largely controlled by geology, land use, catchment and terrain characteristics^16,73,74^. In addition, the meteorological factors, such as high evapotranspiration-induced moisture surplus accelerates a swift recovery. This clearly shows that soil, hydro-meteorological, and terrain features play distinct roles in the propagation of catchment-scale hydrological droughts.

## Discussion and conclusions

The observational evidence indicates strong support that heterogeneity in hydrological drought responses is controlled by feedback between climate-catchment-and-soil attributes (Fig. 4 and Fig. S7). Previous studies^15,16,83,84^ conducted on catchment-scale droughts provide important yet incomplete insights into the role of potential drivers in hydrological drought propagation. Based on an earlier study^85^ that establishes structural control on catchment sensitivity, our approach further expanded on geomorphological features by exploring additional covariates, a range of terrain, and soil characteristics influencing various drought characteristics, which have not been investigated so far—neither in observational assessments nor in land surface model-based simulation^10,86^. The sources of uncertainty in the analyses stem from the quality of available records. Our comparative assessment of the effect of large reservoirs on streamflow droughts over a few selected catchments suggests that streamflow drought tends to become shorter intertwined by a slight increase in drought growth and deficit volume (Fig. S10). Further, while reservoirs escalated the termination rate, no notable changes are apparent for drought recovery. The decline in drought duration and increased termination rate is statistically significant (at 5% significance level; with pvalues 0.015 and 0.021 for the duration and DTR, respectively); however, the increase in growth and deficit volume are not statistically significant, as confirmed by the Wilcoxon rank-sum test. Our findings corroborate with an earlier perspective article^87^, based on several notably large reservoirs across the Globe, which suggests the addition of reservoirs could offset the effect of minor droughts; Still triggers increased low flows and severity of streamflow droughts during prolonged drought episodes due to increased reliance on reservoirs. However, understanding the impacts of reservoirs and their regulations on droughts requires in-depth analyses; for example, hedging policies^88^ for reservoir operations might mitigate the impact of severe water deficits by reserving a certain amount of water in advance for future use. Moreover, climate change may impart nonstationarity in low flow series, which may account for additional uncertainty in the analysis. However, we compensated this by considering average (or median) relationships, which is commonly applied in low flow regionalization studies and followed elsewhere^16^ as a robust measure in presence of weak nonstationarity. Further, accounting nonstationarity in records would require longer hydroclimatic time series, which is limited for the area being considered here.

Our findings have direct implications for catchment-scale drought mitigation. The identified dynamic covariates, such as climate and soil moisture level, could be utilized for monitoring drought stages one to two seasons in advance and to support drought warning efforts by developing a multivariate forecast model, enabling seasonal-to-sub-seasonal (S2S) prediction^89,90^. While meteorological to hydrological drought is forecasted at a monthly to the seasonal time scale in practice^91^, timely issuance of targeted drought early warning systems (DEWS)^92^ and a dynamical low flow forecast at a higher temporal resolution involving primary drought attributes, such as growth, persistence and recovery pattern, could be effective in mitigating impacts. Further, for climatologically heterogeneous regions of India, developing an improved probabilistic S2S low flow forecast, integrating the static and dynamic controls could be of great interest in aiding economic resilience to droughts^93^.

In particular, it is anticipated that a better understanding of the physical processes of detecting drought characteristics will lead to improved water resources management and aid in forecasting efforts during water stress episodes in the future. Unlike several studies that relied on model-simulated records for streamflow drought assessments^94,95^, our observation-based synthesis enables robust risk assessment, effective in impact, adaptation, and vulnerability (IAV) studies in a changing climate. Our study helps resolve the recent debates about drought characteristics over tropical catchments, especially the recovery phase, and highlights the importance of accounting for a holistic approach involving climate-catchment-and-soil feedback to understand drought propagation. The derived insights add value towards risk finance (crop insurance) and early-warning system development, useful for adaptation planning for extreme droughts. Further, we hypothesize that the multistage framework adopted here can be translated to other regions. Furthering this, studies may expand on how the streamflow drought characteristics vary spatially and temporally across different climate types across the Globe. Finally, our observation-based streamflow drought analyses may serve as a basis for climate change impact assessments on catchment-scale drought propagation, especially in tropics^95–97^ and to investigate links between low flows and modes of climate variability^98^.

The obtained insights from this study highlight soil management plays a crucial role in desiccation and its resilience. Since climate variability and change have exacerbated the concurrence of warm-and-dry conditions^99^, the persistence of carbon loss (the “legacy effect”)^100^ a few years after extreme and persistent droughts, may have long-term effects on the carbon-budget of the tropical rain-dominated ecosystem of the Indian peninsula. While soil carbon stocks for peninsular India are relatively low than that of the global average^48^, efficient soil and water conservation measures can improve soil carbon sequestration^101,102^ and enhance drought resilience, ensuring water-and-food security of the country^82^.

## Methods

### Hydro-meteorological forcing data set

We obtain the observed daily streamflow time series from the nationwide water resources information system (India-WRIS; https://indiawris.gov.in/wris/). The land-use pattern reveals an average ~ 16% (ranges from 3 to 33%) area under irrigations considering both surface and groundwater (e.g., tube wells and dug wells) sources^103^. To ensure adequate spatial coverage as well as the completeness of records, we selected the catchments based on the following criteria: (1) The stations with a minimum of 20 years of continuous streamflow record availability during the analysis period (1965–2019); (2) The catchment area of the sub-basin to be at least 1000 km^2^ or more. Based on this criteria, we selected 98 stream gauges with catchment area range between 1200 and 307,800 km^2^ from 18 different river basins across PRB (Fig. 1; Fig. S1). Following the earlier literature^104,105^, we infill the missing gaps in daily streamflow records using the time series interpolation technique.

To examine meteorological control on drought stages, we use the observed gridded meteorological datasets with a spatial resolution of 0.5° available at a monthly time scale. The meteorological variables are precipitation^43^, soil moisture (at 1.6 m depth)^46^, mean air temperature (at a height 2 m above surface)^44^, PET^45^ estimated using the Penman–Monteith method. To identify potential KDDs for catchment-scale drought propagation processes, we obtain catchment boundaries from the Global Streamflow Indices and Metadata (GSIM) archive^47^. To ensure data compatibility, we kept the record lengths of hydrometeorological variables same as the streamflow record lengths for each catchment. Further, the baseflow index for each catchment is calculated following the WMO manual on low-flow estimation procedure^106^.

### Effect of possible flow regulations on streamflow droughts

To assess the effect of flow regulations on regional droughts, we compare drought characteristics of catchments with medium to large-sized reservoirs versus catchments with natural to near- natural flow conditions (Table S2) for representative locations for the common period 1987–2013. Following ref.^81^, we quantify the effect of flow regulations on streamflow droughts for each pair of natural versus the regulated catchments based on relative change statistics,$R_{L} = {{\left( {C_{r} - C_{n} } \right)} \mathord{\left/ {\vphantom {{\left( {C_{r} - C_{n} } \right)} {C_{n} }}} \right. \kern-\nulldelimiterspace} {C_{n} }}$, where *C*_*r*_ and *C*_*n*_ represent the median drought characteristics in regulated and natural catchments, respectively.

### Delineation of drought characteristics

We identify hydrological droughts by applying a variable threshold approach to the daily streamflow time series^15,26,41^. The advantage of using the variable threshold method of drought delineation over the constant threshold is two folds: (1) Ability to capture the seasonal variability that prevents the natural low flow season to be detected under drought (2) enables detections of various drought characteristics rather than instantaneous drought onset and termination points as followed in the standardized index-based drought detection approach (e.g., standardized indices of precipitation^107^ and streamflow^108^). For the threshold determination, 366 (an additional day for leap year) flow duration curves are developed using continuous time series of streamflow records. Following the literature^15,16,109,110^, an 20th percentile threshold (flow equaled or exceeded 80% of the flow record) is selected for each day of the year forming the variable threshold time series. Since the daily threshold time series appeared to be a jagged curve resulting in several short deficit periods, a centered moving average of 30 days is applied as a smoothing filter^26,41^. A drought episode is detected when the daily streamflow time series falls below the variable threshold.

After identifying drought events, next, we further categorize streamflow-based droughts into several characteristics^36,41^ (see Fig. 1b). Drought duration is the period in which streamflow is lower than the threshold continuously for 30 days or more (this phase is shown from *t*_*sp*_ to *t*_*ep*_ in Fig. 1b, where ‘*s*’ denotes initiation, ‘*e*’ is the termination point and ‘*p*’ indicates persistence phase). Following Ahmadi and Moradkhani^41^, we select the threshold time window of 30 days based on the consideration of the natural variation and long enough to filter out the inter-seasonal anomalies. Following the refs.^36,37,41,^ we detect the drought growth as moving 60 days back from the drought termination, when the streamflow falls above the threshold for less than 15 days, i.e., the occurrence of short deficits interrupted by less than 15 days of above-normal streamflow (in Fig. 1b: t_sg_ to t_eg_, where ‘s’ is the initiation, ‘e’ is the termination, and ‘g’ denotes the growth). We detect the recovery period as moving 60 days forward from the end of the persistence phase, when the streamflow falls below the threshold for less than 15 days (in Fig. 1b: t_sr_ to t_er_ where ‘s’ is the initiation, ‘e’ denotes the termination and ‘*r*’ shows the recovery phase). If the streamflow time series persistently remains below the threshold for more than 15 days then we mark ‘no recovery’ and the following episode is then considered as a part of a multi-season drought event. Finally, we quantify DTR as the magnitude of change in flow from the Maximum Drought Deficit volume (MDD, the day with the largest negative departure from normal streamflow between the time of the start of drought development and the time of the end of drought termination in Fig. 1b—for details please see last but one paragraph in page 4267 in Parry et al.^36^) to the peak surplus flow (PS, Fig. 1b), divided by the time taken for this transition.

We determine the seasonality in drought termination using directional (or circular) statistics. The termination date is used as a directional variable^50^ (SI 1.1), in which the position of the mean termination date can be determined using angles (Eq. 1.3 in SI 1.1). Following the ref.^111^, we calculate the mean termination day (i.e., mean direction of the day of drought termination as described by the circular data) and its variance by weighing the deficit volume (see SI 1.1), ensuring the events are given importance as per the persistency of the event.

### Determination of base flow and topographical wetness index

Baseflow is the slowly varying portion of streamflow, originating from groundwater storage and/or delayed sources such as channel bank storage, lakes, wetlands and melting snow and ice^68^. Baseflow is one of the important low-flow hydrological characteristics in semi-arid environments, which is a function of several catchment properties, such as topographic, geologic, soil, and climatic properties^65,68,71^. While for tropical catchments of PRB, contribution from melting snow and ice can be neglected, baseflow is the primary source of water for streams during the periods with little to no precipitations. Baseflow is influenced by sub-surface characteristics, surface elevational gradient, soil depth, and the permeability of geologic and geomorphic features that control deep water storage^112^. The BaseFlow Index (BFI) is the ratio of long-term mean baseflow volume ${(V}_{base})$ to the total streamflow volume (*V*_*total*_) and expressed through the following relation

1 $$BFI=\frac{Base\,flow\,volume}{Total\,flow\,volume}=\frac{{V}_{base}}{{V}_{total}}$$

The BFI has a strong relationship with climate and geology and it controls the catchment-scale drought propagation^16,17,74^. The value of BFI varies from near 0 to 1. A value close to 0 indicates a river has a low proportion of baseflow, e.g., a river with greater peak flow and short lag time, and characterized by impermeable geology with little groundwater contribution. In contrast, a BFI value close to 1 has a high proportion of baseflow, e.g., a stable river with relatively permeable geology with substantial groundwater contribution. In periods of dry weather when streamflow is significantly reduced (as in PRB), rivers with high BFI values indicate groundwater inflow sustaining stream flows^71^.

We calculate the Base Flow Index (BFI) based on the method described in WMO Manual on low-flow estimation and prediction^106^: (i) we divide the daily streamflow values (m^3^/s) into five days non-overlapping block and selected the minimum flow value, *Q*_*m*_ from each block. (ii) Identify the turning point, *Q*_*t*_ from the sequence of *Q*_*m*_ values that satisfies the condition that if 0.9 × central value ≤ adjacent value, then central value becomes the turning point. (iii) Next, join the *Q*_*t*_ values, and perform a linear interpolation between two turning points to get base flow for each day. (iv) Finally, we determine the BFI by dividing the amount of streamflow volume beneath the baseflow line to the total amount of water beneath the hydrograph. Our obtained BFI values are well within the range of the global map of median estimated BFI in ref.^68^, which shows for peninsular Indian catchments the BFI values vary between 0.2 and 0.75. We illustrate the computation of the BFI from daily streamflow records for a selected catchment, i.e., Anandapur gauged site in Baitarani River Basin, located at eastern India, for the period of available records, 1973–2018 (Fig. S11). While the BFI value for the whole period is 0.45, the annual BFI value, which is determined by summing up the base flow and total volume separately for the year 1990, shows a value of 0.48. Overall, a low BFI value for the Anandapur catchment suggests an impermeable catchment with a steep rising limb of the hydrograph characterized by a small lag time.

Topographic Wetness Index (TWI) quantify the effect of local topography on hydrological processes^113^ and was developed by Beven and Kirkby^75^. It is defined as, $TWI = \ln \left( {{{A_{s} } \mathord{\left/ {\vphantom {{A_{s} } {\tan \,\beta }}} \right. \kern-\nulldelimiterspace} {\tan \,\beta }}} \right)$, where $A_{s}$ denotes the specific catchment area (*i.e.*, catchment area divided by the cell width in slope direction; in m^2^ m^−1^) and $\beta$ indicates the local slope in the steepest down slope direction of the terrain (in radians)^114^. The TWI for this study was determined from the 90 m shuttle radar topographic mission (SRTM) digital elevation model^115^ using the System for Automated Geoscientific Analyses (SAGA v.6.3.0) software^116^.

### Digital soil mapping (DSM)

We develop Digital soil maps primarily for nine different soil parameters, e.g., sand and clay contents, SOC contents, SOC stock, pH, CEC, moisture contents at field capacity and permanent wilting point, and available water capacity for the Indian subcontinent at six standard depths (0–5, 5–15, 15–30, 30–60, 60–100, and 100–200 cm respectively) according to the GlobalSoilMap specifications^117^. We develop DSMs using an Indian soil legacy database that utilized archived data from various sources, such as the National Bureau of Soil Survey and Land Use Planning (NBSS&LUP) and other institution publications^18^. The newly developed, digital soil map follows *scorpan* model^118^, in which a soil property at an unknown location is estimated as a function of environmental covariates. The environmental covariates used in generating the current maps include terrain attributes derived from the 90 m shuttle radar topographic mission (SRTM) digital elevation model (DEM) data^115^ and climate covariates, which includes mean monthly temperature and precipitation^18^.Soil parameters (Table S1) for top 30 (weighted average of depths 0–5, 5–15, 15–30 cm) and 100 cm (weighted average of depths 0–5, 5–15, 15–30, 30–60, 60–100 cm) soil layers are extracted over the selected catchments of PRB.

### Linking drought stages with climate-catchment-soil controls

To identify the potential KDD in influencing drought dynamics, first we perform a non-parametric correlation analysis. Table S1 lists all 89 covariates that are chosen to identify key drought drivers (KDD). Among climatological attributes, we also consider several hydro-meteorological indices, especially for extremes calculated from monthly time series of precipitation (Rainfall_20p), temperature (TX90p), PET (PETX_20p), and soil moisture (SMX_20p), which are widely used for analysing climatic extremes at the regional and global scales^119,120^. These extreme indices are determined by calculating the median of the values greater (or lower) than equal to the *n*th percentile (where, *n* = 20 for deficit and 90 for surplus as adopted here) of each meteorological variable. Next, we perform dependency analysis between each KDD and catchment-wise median drought stages using Kendall’s τ, which is robust to the small number of outliers (unlike Pearson’s correlation coefficient) and discrepancies in the data^121^. We check the statistical significance of dependence at 10% significance level with *p*-value < 0.1.

Finally, to select KDD influencing the drought stages, we implement a hybrid feature selection procedure consisting of filtering and wrapping through Boruta algorithm^78^, which is built around the random forest classification algorithm. For filtering, we retain the covariates exhibiting significant (*p*-value < 0.1) association with drought stages in the Kendall’s rank correlation. Subsequently, we apply Boruta on the reduced set of significant variables to obtain the key drought drivers by fixing the number of iterations as 1000 (Fig. 1c). This was achieved by creating ‘shadow’ attributes for each original attribute from shuffling the corresponding values of original covariates across stations. Finally, we perform feature selection by using the random forest classification algorithm and compute the importance of all attributes of this extended system with reference to maximum Z-score of shadow attributes (MZSA). We mark the variables significant when they have ‘importance’^78^ significantly higher than that of MZSA and discard those variables that show ‘importance’ lower than that of the MZSA.

## Supplementary Information

Supplementary Information.

## Data Availability

All the data used in this study are publicly available. The precipitation data is obtained from Global Precipitation Climatology Centre (https://opendata.dwd.de/climate_environment/GPCC/html/fulldata_v7_doi_download.html). The monthly soil moisture data is obtained from the Climate Prediction Center (CPC; https://psl.noaa.gov/data/gridded/data.cpcsoil.html). The monthly mean surface air temperature is obtained from the CPC Global land surface air temperature data (https://ual.geoplatform.gov/api/items/ff4f9af65d322c28a421cf569471d216.html). The PET time series is obtained from the Climate Research Unit’s (CRU) version 4.04 database (https://crudata.uea.ac.uk/cru/data/hrg/). All data are available at a 0.5° spatial resolution in a monthly time scale. The shapefiles for the Indian river basins are obtained from the Global Streamflow Indices and Metadata Archive (https://doi.pangaea.de/10.1594/PANGAEA.887477). The digital elevation map to develop terrain features are derived from the 90 m SRTM DEM database (https://cgiarcsi.community/data/srtm-90m-digital-elevation-database-v4-1/). The digital soil mapping for India was developed using an Indian soil legacy database that utilized archived data from various sources, such as the National Bureau of Soil Survey and Land Use Planning (NBSS&LUP; https://www.nbsslup.in/) and other institution publications^18^.

## Code availability

The MATLAB Codes used for drought characteristics and delineation of drought regimes have been archived by the authors and are available on request from P.G., pganguli@agfe.iitkgp.ac.in. The source codes for Digital Soil Map of India codes are available from authors through personal request.
