# Supplementary material for: Climate-catchment-soil control on hydrological droughts in peninsular India
Source: Sci Rep. 2022 May 15;12:8014. doi: 10.1038/s41598-022-11293-7 (PMC9108094; doi:10.1038/s41598-022-11293-7)
Supplement: Supplementary file 1 — Supplementary Information. [file 41598_2022_11293_MOESM1_ESM.pdf]

Supplementary Information for

Climate-Catchment-Soil Control on Hydrological Droughts in Peninsular India

Poulomi Ganguli[1,*], Bhupinderjeet Singh[1], Nagarjuna N. Reddy[1], Aparna Raut[1], Debasish Mishra[1], Bhabani Sankar Das[1]

[1]Agricultural and Food Engineering Department, Indian Institute of Technology Kharagpur, West Bengal, Kharagpur 721302, India

[*]Corresponding Author: pganguli@agfe.iitkgp.ac.in

SI. 1.1 Determination of Seasonality in Drought Termination

The termination date of each drought event is plotted on the circle with unit radius, where the position of the event is defined by θ_i

$$\theta_i = \frac{2\pi * D}{T} \tag{1.1}$$

where T is the number of days in the year, D is the termination date which varies from 1 to 365 days in a non-leap year (366 days in a leap year), The position of the mean termination date can be determined using the angles, converting it to x and y coordinates:

$$\overline{x} = \frac{\sum_{i=1}^{n} q_i \cos \theta i}{\sum_{i=1}^{n} q_i} \qquad \overline{y} = \frac{\sum_{i=1}^{n} q_i \sin \theta i}{\sum_{i=1}^{n} q_i} \tag{1.2}$$

where q_i = Deficit volume for the event 'i'

The mean direction of the circular Data ($\overline{\eta}$) is determined as:

$$\overline{\eta} = \begin{cases} \tan^{-1}\left(\frac{\overline{y}}{\overline{x}}\right) & if\ \overline{x} > 0\ and\ \overline{y} > 0 \\ 180 - \tan^{-1}\left(\frac{\overline{y}}{\overline{x}}\right) & if\ \overline{x} < 0\ and\ \overline{y} > 0 \\ 180 + \tan^{-1}\left(\frac{\overline{y}}{\overline{x}}\right) & if\ \overline{x} < 0\ and\ \overline{y} < 0 \\ 360 + \tan^{-1}\left(\frac{\overline{y}}{\overline{x}}\right) & if\ \overline{x} > 0\ and\ \overline{y} < 0 \\ \pi/2 & if\ \overline{x} = 0\ and\ \overline{y} > 0 \\ 3\pi/2 & if\ \overline{x} = 0\ and\ \overline{y} < 0 \end{cases} \tag{1.3}$$

Mean Event Date can be calculated as: $\omega = \tan^{-1}(\overline{\eta})\left(\frac{len\,\overline{y}}{2\pi}\right)$

Where ω is the mean date of occurrence of the extreme events and $\overline{\eta}$ is computed using Eq. 1.3. $len\,\overline{y}$ indicates the average length of days in a year, considering the number of leap and non-leap days. Finally, to measure the variability in the termination month about mean date is calculated by defining the regularity, $\overline{\phi}$:

$$\overline{\phi} = \sqrt{\overline{x}^2 + \overline{y}^2} \qquad 0 \le \overline{r} \le 1 \tag{1.4}$$

Where, $\overline{\phi}$ = 0 if all the events are terminating uniformly throughout the year (low regularity) and $\overline{\phi}$ =1 if all the events are terminating in the same month (high regularity)

The variability in timing of drought termination is determined using circular variance (s^2):

$$s^2 = -2\ln\left(\bar{\phi}\right) \tag{1.5}$$

SI 1.2 Drought Cluster Identification using Fuzzy Algorithm

Fuzzy C- means (FCM) algorithm was firstly proposed by which was further improved[1,2]. The FCM algorithm assigns the membership to each feature vector with respect to the euclidean distance between the feature vector and cluster center, and it is more generalized and useful to describe a point not by a hard clustering, but by its membership values with respect to all the clusters[3]. The higher the value of fuzzy membership stronger is the relationship of the feature vector with the specific cluster[4]. For a data set of *M* objects and *p* classes, if $\mathbf{X}_k$ is the feature vector of the kth object, where k = 1,2, 3…., *M*, the main aim of the FCM algorithm is to minimize the objective function as defined below:

$$\text{J (U, C)} = \sum_{j=1}^{M}\sum_{i=1}^{c} u_{ik}^{a}\, \|Y_k - C_i\|^2 \tag{1.6}$$

Where, u_{ik} is the membership value of kth data point in the ith cluster, $\|Y_k - C_i\|^2$ is the Euclidean distance between feature vector k and a center point of ith cluster, C_i is the center value of the ith cluster and α is called as fuzzifier value, which can have any value which is greater than 1. The value closer to 1 provides the cluster solution which is very similar to hard clustering (*e.g.*, K-means clustering) algorithm. In general, fuzzifier value ranges from 1 to 2.5[5].

Fuzzy c-means Algorithm Steps:

1. The number of clusters and the data vector of the cluster center is assumed at random.

2. Membership value matrix is calculated using Eq. (1.6)

$$u_{i<k}^{t+1} = \left[\sum_{j=1}^{c}\left[\frac{\|y_k - c_i\|}{\|y_k - c_j\|}\right]^{\frac{2}{\alpha-1}}\right]^{-1} \tag{1.7}$$

Where i= 1,2,…...*c*, k=1,2,…,*M*. j = *1,2.,….c*

3. Using the updated membership values and equation, new values for the cluster center are calculated as below:

$$C_i = \frac{\sum_{k=1}^{M} u_{ik}^{a} y_k}{\sum_{k=1}^{M} u_{ik}^{a}} \tag{1.8}$$

Finally, the clustering process is stopped when it follows a certain stopping criterion. For our case, we stopped the clustering process when two successive iterations reached a value of objective function less than 0.001.

Table S1. List of covariates selected to identify key drought drivers

Attribute types	Specifics of attributes	Abbreviations	Units
Soil	Clay content at 30, 100 cm depth*	Clay_30[1], Clay_100[2]	%
	Sand content at 30, 100 cm depth	sand_30, sand_100	%
	pH at 30, 100 cm depth	pH_30, pH_100	-
	Soil organic content at 30, 100 cm depth	SOC_30, SOC_100	%
	Cation exchange capacity at 30, 100 cm depth	CEC_30, CEC_100	cmol/kg
	Stock at 30, 100 cm depth	Stock	t/ha
	Field capacity at 30, 100 cm depth	FC	%
	Permanent Wilting Point at 30, 100 cm depth	PWP	%
	Available Water Content at 30 cm, 100 cm depth	AWC	%
	Annual average soil moisture, median monthly soil moisture ≤ 20th percentile threshold	Mean_SM, SMX_20	mm
Climate	Annual average rainfall, mean monthly rainfall from January – December, median monthly rainfall ≤ 20th percentile threshold	Rainfall_*i*, where, *I* = 1, 2,…., 12; RM_20	mm
	Annual average potential evapotranspiration, mean monthly potential evapotranspiration, and median monthly potential evapotranspiration ≤ 20th percentile	Mean_PET, PET_*i* where, *I* = 1, 2,…., 12; PETX_20	mm/day
	Annual average monthly temperature, mean monthly temperature, and median monthly temperature ≥ 90th percentile	TM, TM_*i* where, *I* = 1, 2,…., 12; TX90	mm
Catchment	Aspect		radian
	Channel network base level	CNBL	m
	Convergence Index		-
	Cross-sectional curvature		m^{-1}
	Elevation		m
	Flow accumulation		m^2
	Hill shading		radian
	Longitudinal curvature		m^{-1}
	Slope length-gradient factor	LS-factor	-
	Relative slope position		-
	Slope		radian
	Terrain ruggedness index		-
	Topographic wetness index		-
	Valley depth		m
	Vertical distance to channel network	VDCN	m

*0-30 cm depth indicates the top soil, 30-100 cm indicates the sub-soil; the superscripts, 1 and 2 indicate the 30 cm and 1 m depths respectively.

Table S2. Details of catchments to assess effect of flow regulations on streamflow droughts

Types of Catchments	Sr.#	Basin	Sub-Basin	Stream gauge Location		Reservoir Name	Reservoir Location[2]		Reservoir Capacity (BCM)
				Latitude (°)	Longitude (°)		Latitude (°)	Longitude (°)	
With Reservoir	1	Narmada	Mandleshwar	22.17	75.66	Indira Sagar	22.19	75.65	4.42
	2	Krishna	Wadenapalli	16.79	80.13	Nagarjuna Sagar	16.52	79.22	4.08
	3	Mahanadi	Tikarapara	20.60	84.78	Hirakud	21.63	83.81	3.92
	4	Krishna	Huvinhedgi	16.49	76.92	Almatti	16.35	75.73	1.61
Without Reservoir	1	Mahanadi	Basantpur	21.722	82.79	-	-	-	-
	2	Godavari	Nowrangpur	19.198	82.51	-	-	-	-
	3	Godavari	Somanpally	18.620	79.81	Singur	17.76	77.91	0.04*
	4	Penniyar	Vazhavachanur	12.067	78.98	-	-	-	-

*Small reservoir, based on classification given in ref.[6]. The reservoir details are obtained from India-WRIS website: https://indiawris.gov.in/wris/#/Reservoirs

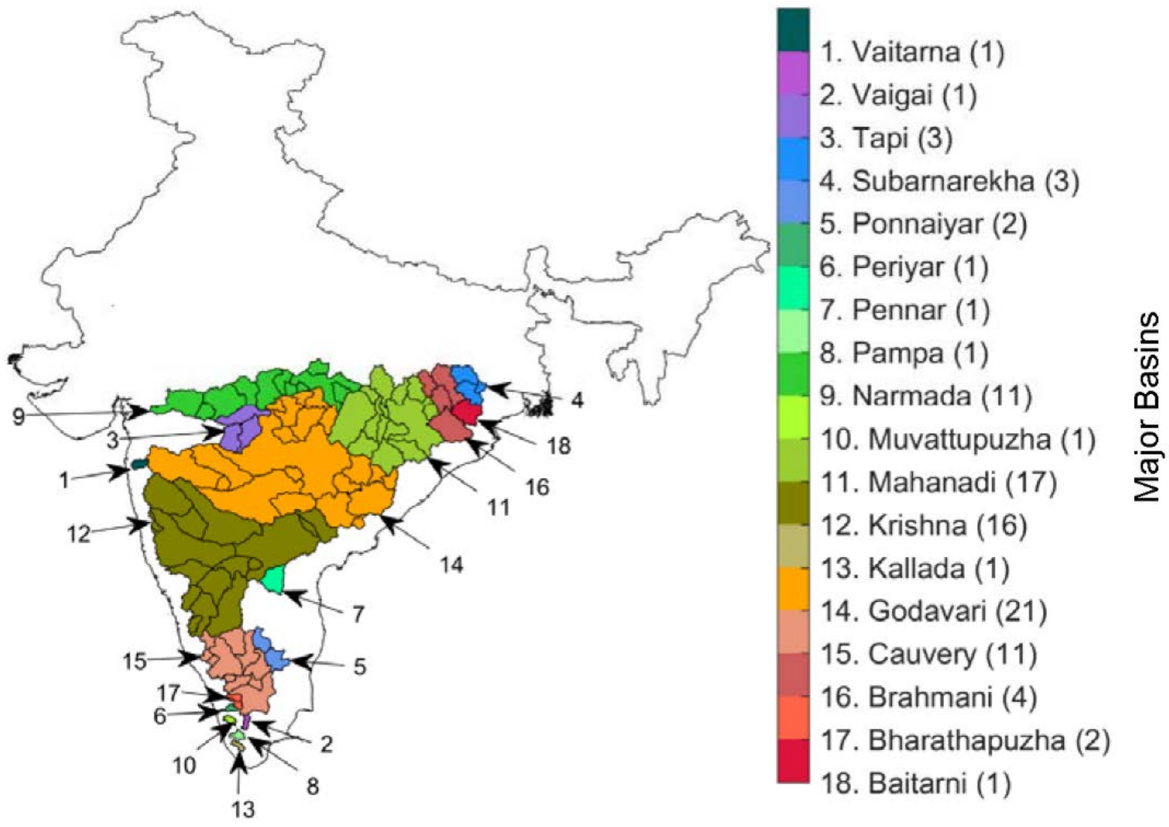

Fig. S1 Locations of large river basins. The numerals in parentheses show the number of sub-catchments within each river basins. The figure is prepared in MATLAB R2020b (academic version) [Software].

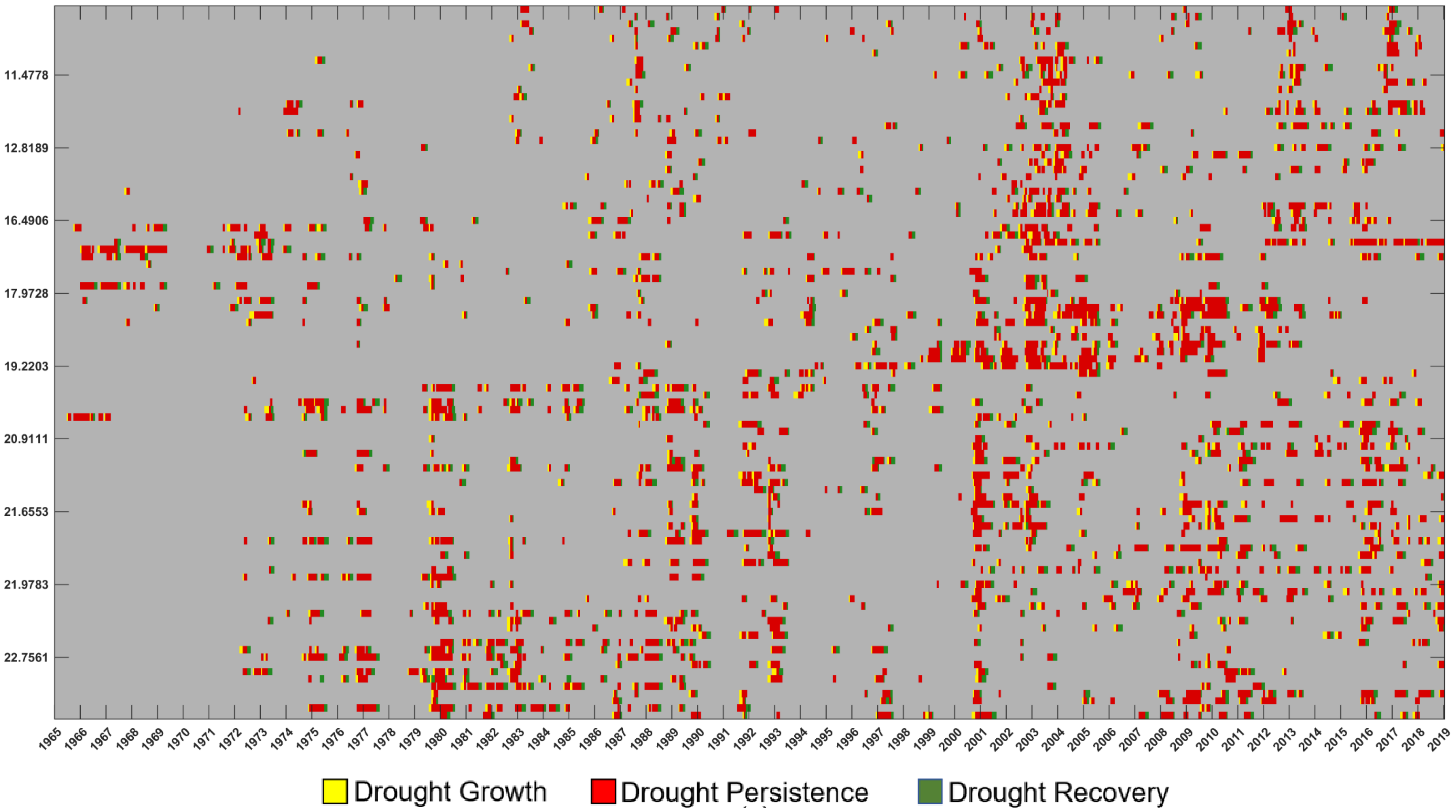

Fig. S2 Hovmöller diagrams (time vs latitude sections) of drought characteristics for the period between 1965 and 2019 over the 98 Peninsular Indian Catchments. The figure is prepared in MATLAB R2020b (academic version) [Software].

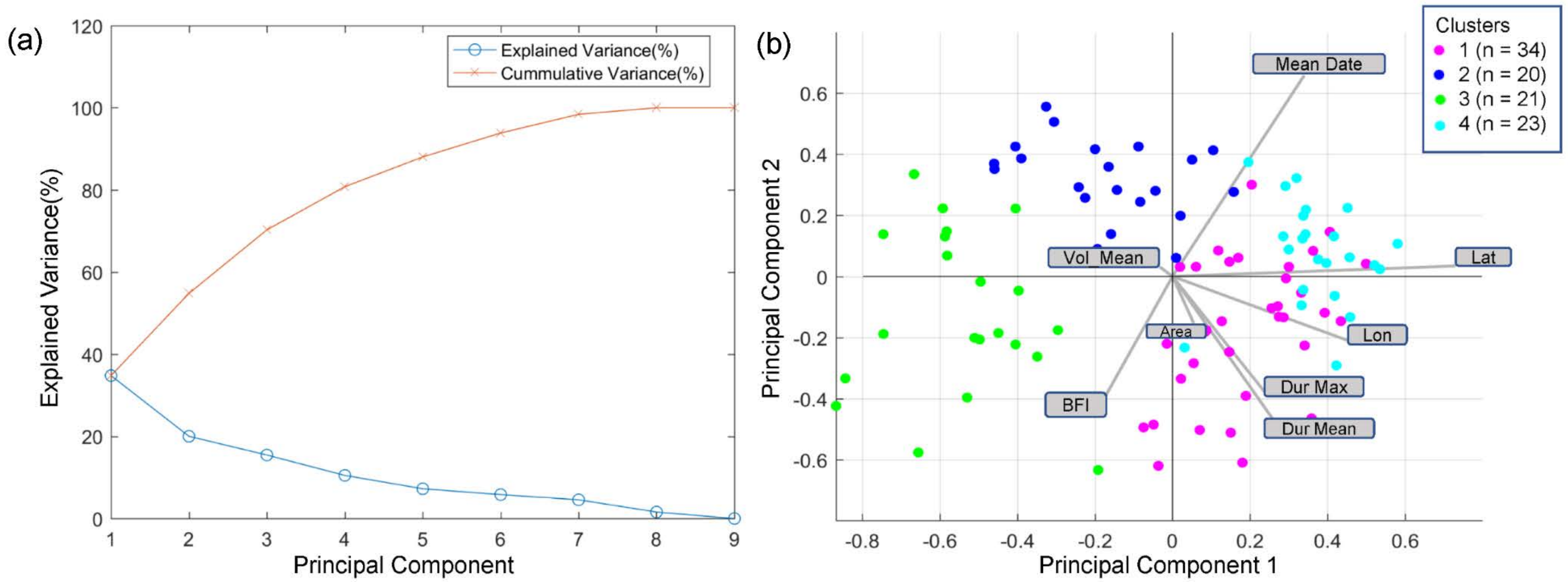

Fig. S3 Identification of drought cluster based on climate and catchment characteristics (a) Explained variance by different principal components (PCs) b) Biplot of the principal components (PCs). Colors indicate the cluster of the catchment. Dur Max, Dur Mean and Vol Mean denote the maximum duration, mean duration and the mean deficit volume respectively. Grey arrows indicate the loadings of the original catchment attributes in the PCA space. The symbol, n in the legend shows the number of sites considered in each cluster. All selected attributes are rescaled and transformed between 0 and 1 using the standard normalization $(\mathrm{X(i)} - \mathrm{minimum}\,(\mathrm{X}))/\mathrm{Range(X)}$, where X indicates selected attributes and $\mathrm{X}(i)$ denotes the attribute value corresponding to each site) before the PCA operation, ensuring the values of the attributes are within the same range. The figures are prepared in MATLAB R2020b (academic version) and then organized in MS Office Power point 2016 [Software].

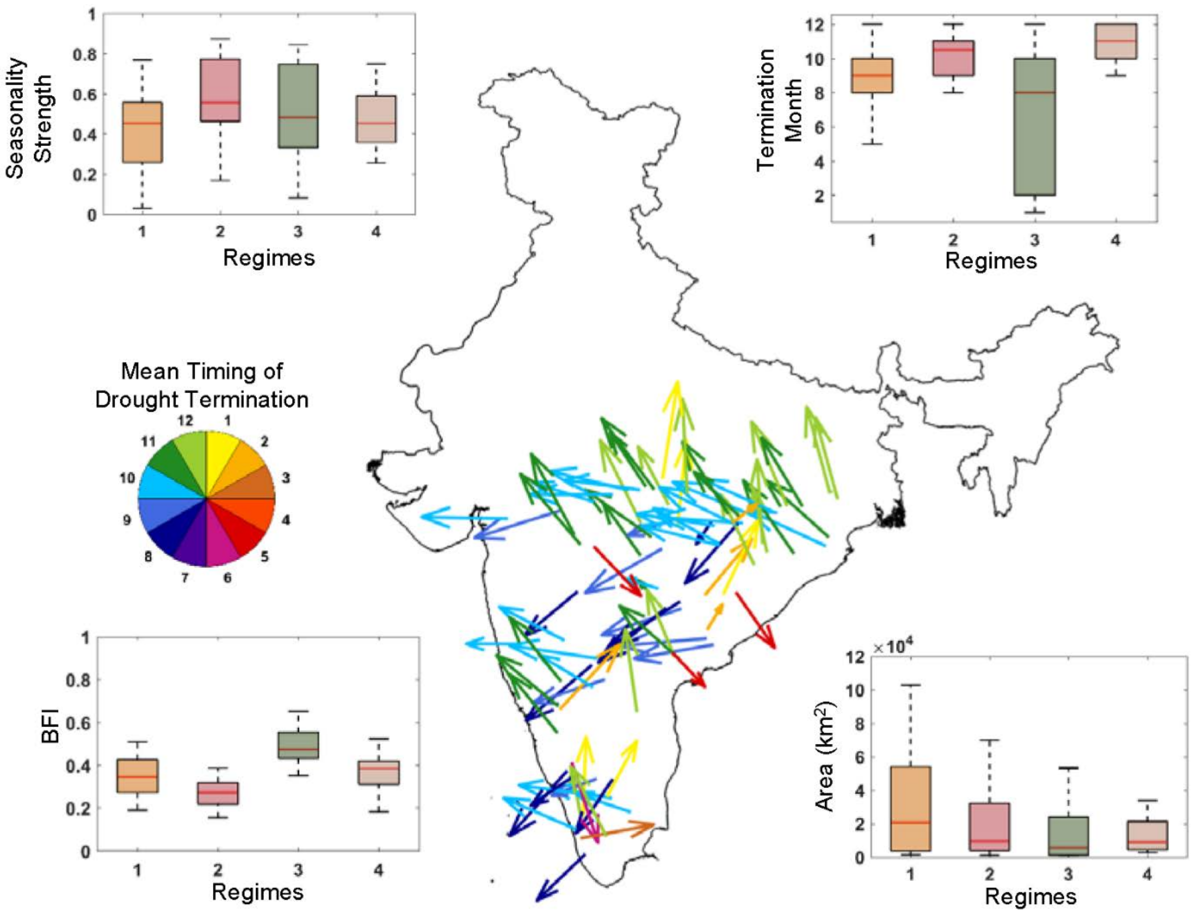

Fig. S4. Spatial distribution of seasonality in drought termination and catchment-specific attributes depicting each region. The shade of the arrow with direction shows the mean timing of drought termination. The length of the arrow shows the circular variance (s^2) for each station; the larger (small) is the size of the arrow, the more (less) is the variability. The boxplot depicts the regional share of seasonality strength (or regularity in drought termination), average termination months, the baseflow index, and catchment area. The shades in the boxplot represent each region. The pie chart (on left) shows the mean timing of drought termination. The shades in the pie chart show the mean termination month: For example, '1' denotes January, whereas '12' indicates December. The figures are prepared in MATLAB R2020b (academic version) and then organized in MS Office Power point 2016 [Software].

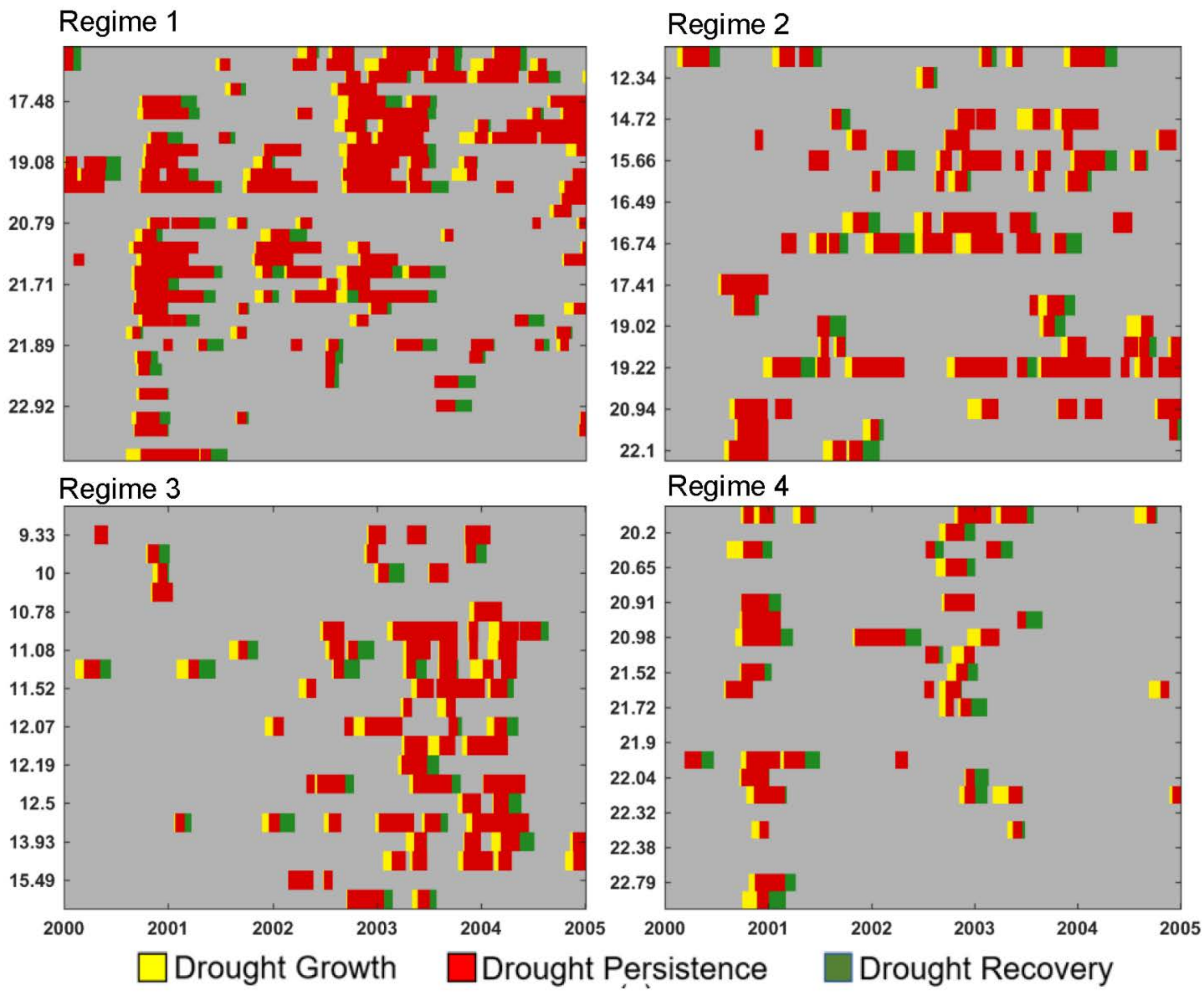

Fig. S5 Hovmöller diagrams (time vs latitude sections) of drought characteristics showing two major historical drought episodes 2000-01 and 2003-04 spanning in the historical time window 2000-05. The figures are prepared in MATLAB R2020b (academic version) [Software].

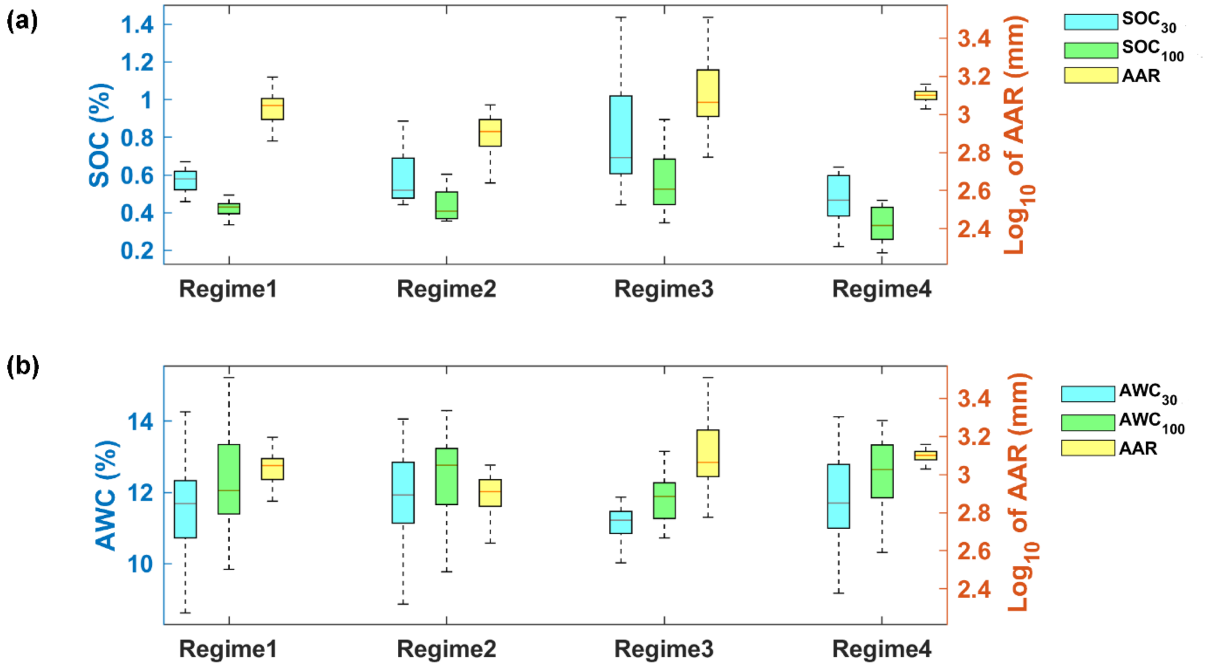

Fig. S6. Regional distribution of soil organic carbon content versus available water. (a) Soil organic carbon (SOC; in percentage) at the surface (30 cm depth) and sub-surface (100 cm depth) levels versus annual average rainfall at a logarithmic scale. (b) Surface and sub-surface available water contents (AWC; in percentage) versus annual average rainfall at a logarithmic scale. The variables in the boxplot SOC_{30} and SOC_{100} denote the soil organic contents at 30 and 100 cm depths, whereas AWC_{30} and AWC_{100} indicate the available water contents (soil water storage capacity) at 30 and 100 cm depths, respectively. The figure is prepared in MATLAB R2020b (academic version) [Software].

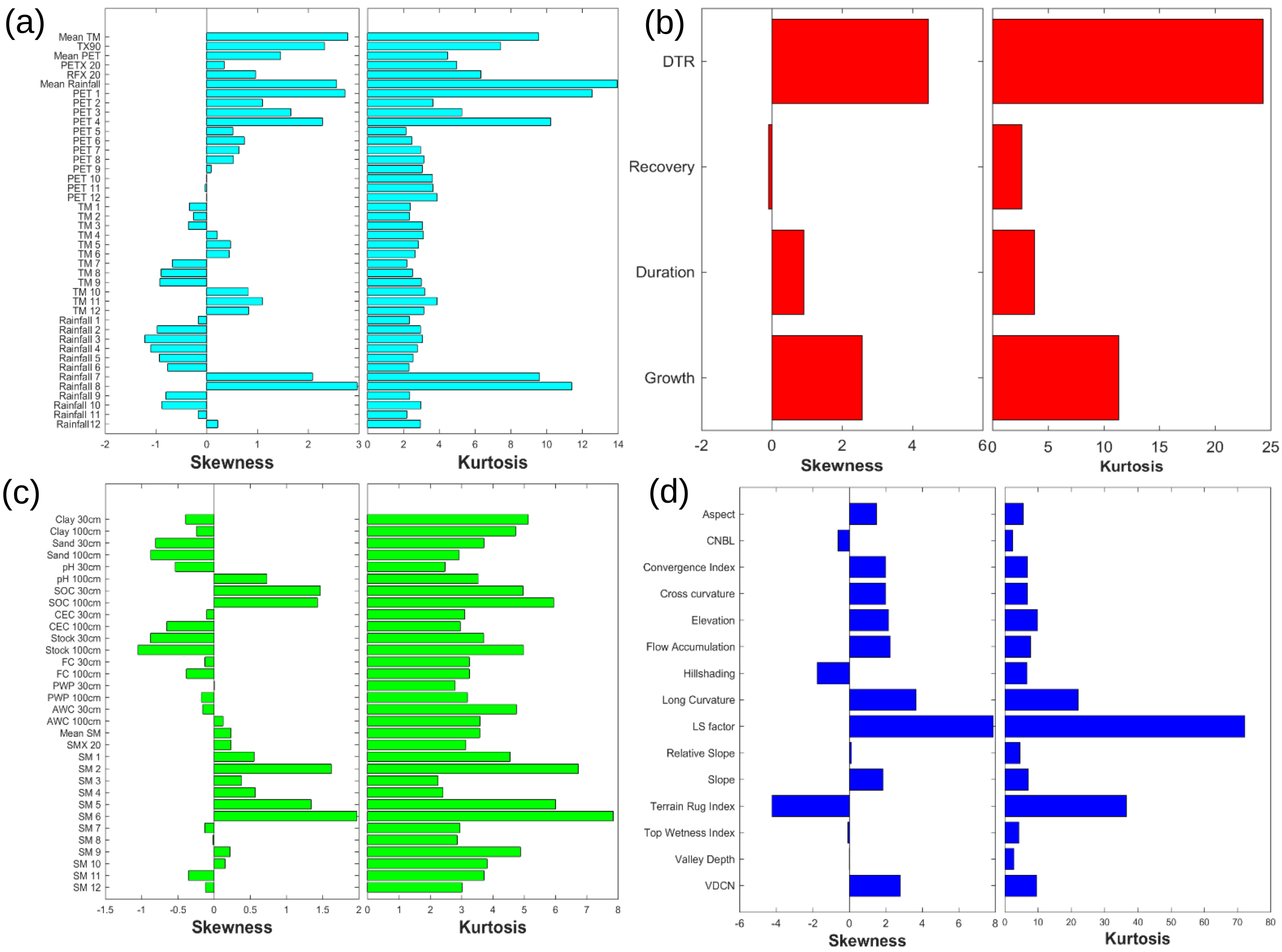

Fig. S7. Skewness and kurtosis values for **(a)** meteorological variables **(b)** drought properties **(c)** soil properties **(d)** catchment characteristics. The figures are prepared in MATLAB R2020b (academic version) and then organized in MS Office Power point 2016 [Software].

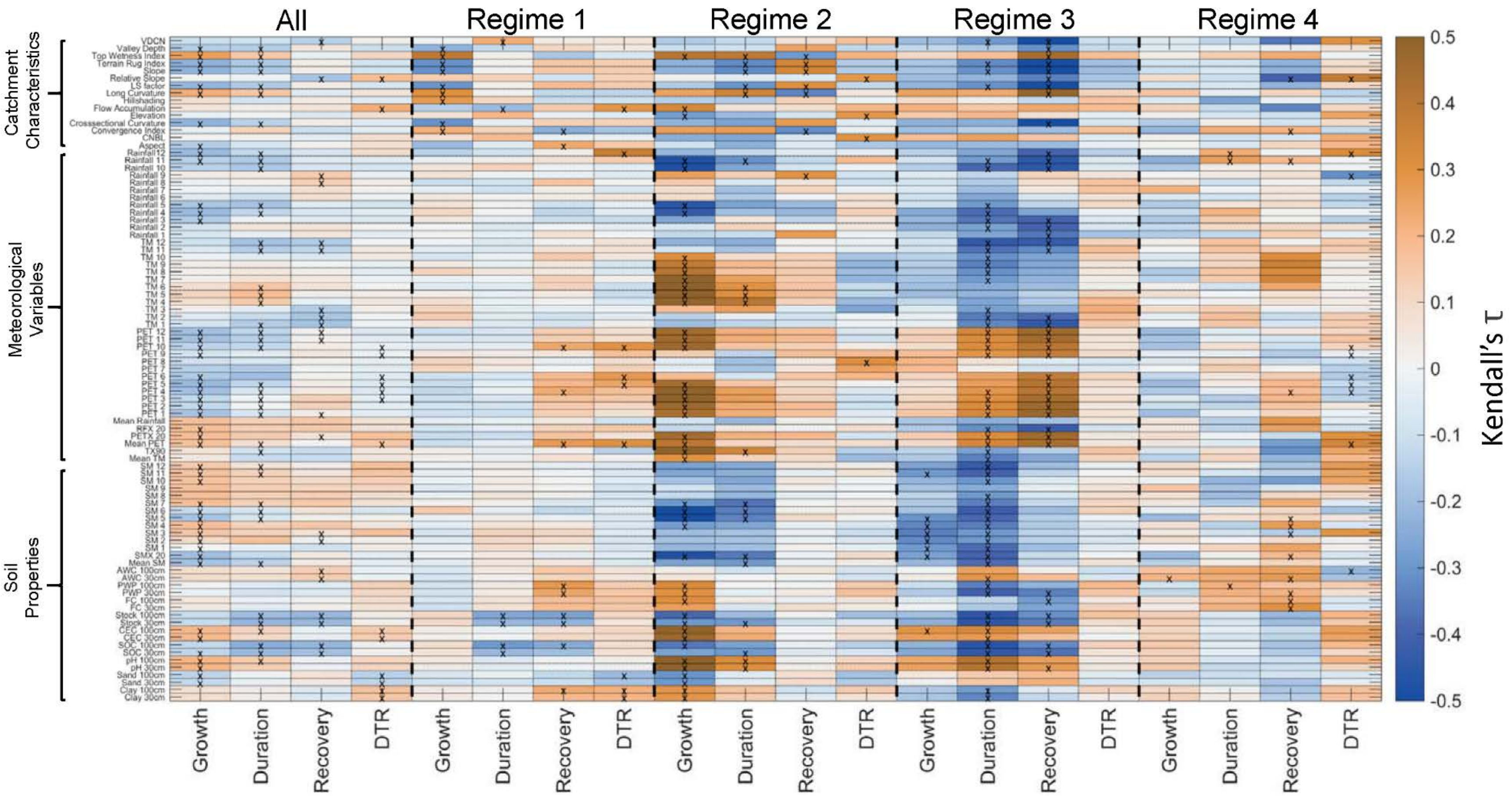

Fig. S8. The Kendall's tau correlation values of 89 covariates as listed in **Table S1** with four different stages of drought namely; growth, duration, recovery and drought termination rate. The crosses show the significant correlation between the variables at 10% significance level. The figure is prepared in MATLAB R2020b (academic version) [Software].

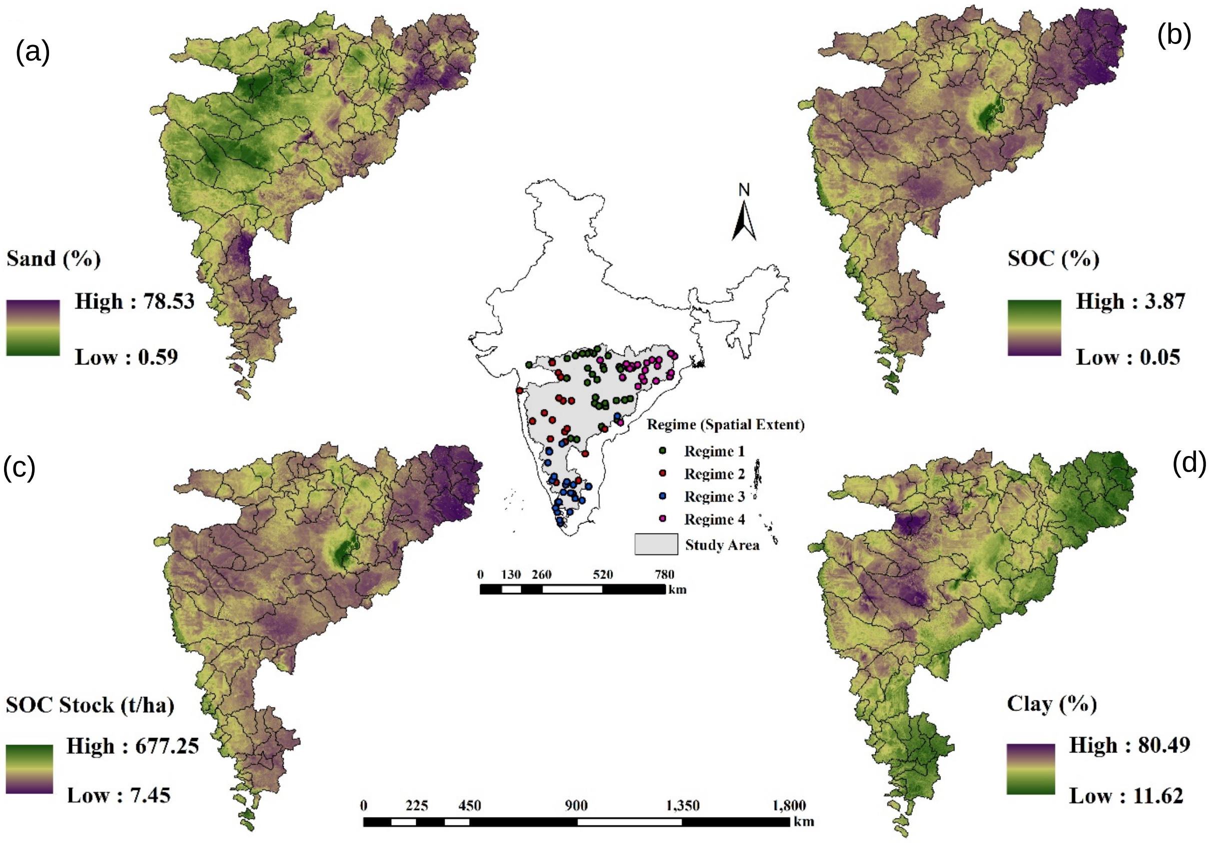

Fig. S9. **Maps for selected soil KDDs as obtained from Boruta feature selection algorithm for various drought stages (a)** average drought growth period; **(b)** average drought duration; **(c)** average drought recovery and **(d)** average drought termination rate attributes across 98 catchments. The India map in the middle shows the delineated drought regimes over the PRB. The map is developed by applying a three-dimensional random forest method coupled with a spatial statistics tool, kriging. The spatial resolution of the map is 500 m. The soil properties mapped in subplots (**a**-**d**) are obtained from the newly developed digital soil maps for India in Reddy *et al*[7]. The shapefiles for the Indian River basins are obtained from the Global Streamflow Indices and Metadata Archive (https://doi.pangaea.de/10.1594/PANGAEA.887477). The locations of the stream gauges are obtained from the India-WRIS archive (https://indiawris.gov.in/wris/#/). The figures are prepared in R-4.0.5 (64 bit) windows version (subplots **a-d**), ArcGIS 10.1 (the India map, locations of stream gauges, and the shapefiles of river basins), and then organized in the MS Office Power point 2016 [Software].

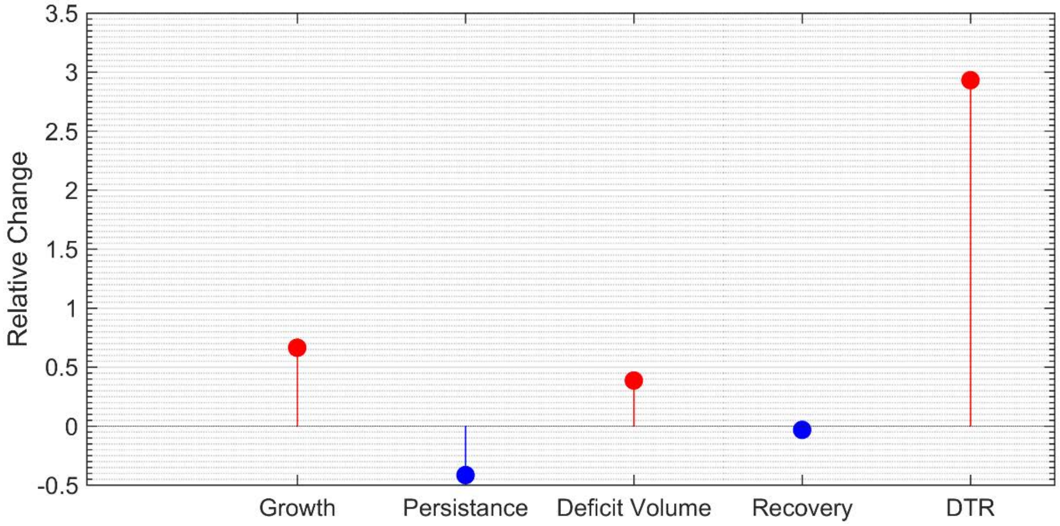

Fig. S10. Effect of reservoir on streamflow droughts. The vertical bars show changes in drought characteristics for catchments with major reservoirs versus natural to near-natural catchments. The bars in red show an increase, whereas the one in blue indicates a decrease in relative change statistics. The figure is prepared in MATLAB R2020b (academic version) [Software].

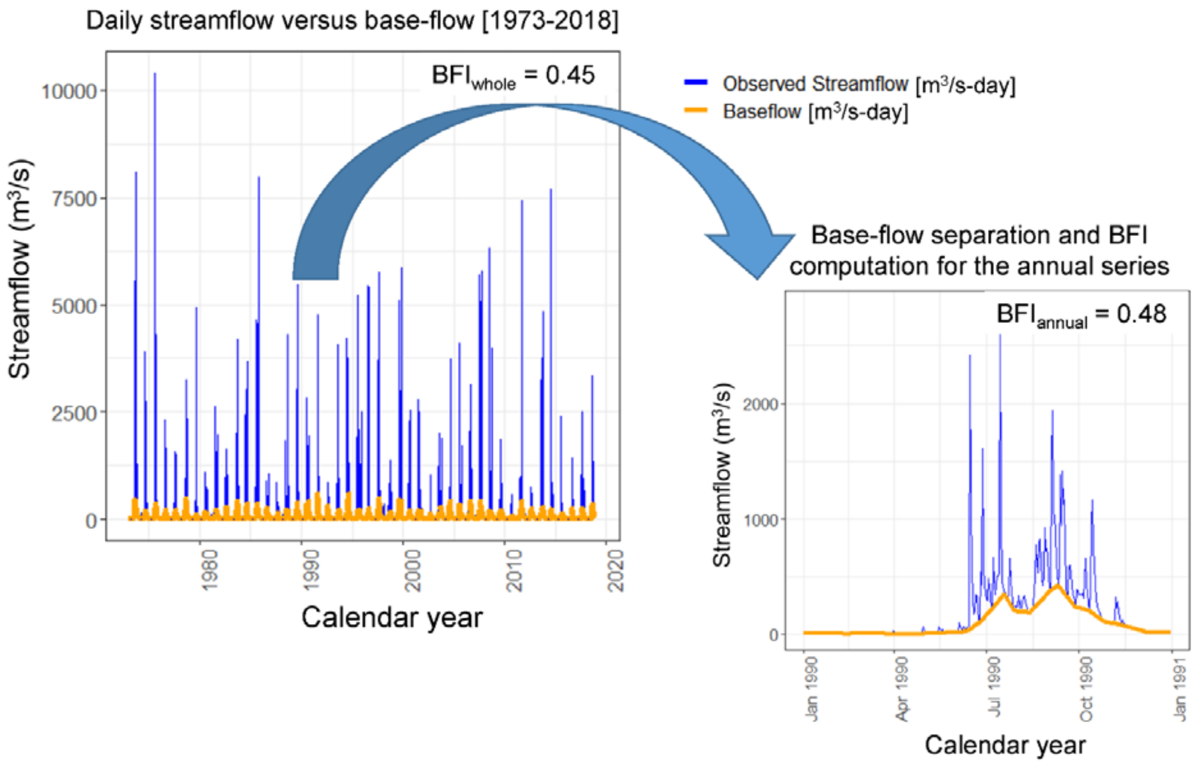

Fig. S11. Illustration of base-flow separation and BFI computation for a representative catchment. The base-flow time series of the Anandapur catchment in the River Baitarani from 1973 to 2018 is delineated based on the method described in the WMO Manual on low-flow estimation and prediction[8]. The BFI index, BFI_{whole} for the entire period, is determined by the volume of the base flow to the total flow for the whole period (1973-2018). The inset shows the annual BFI estimates for the calendar year 1990 (from 1st January – 31st December) indicating the index's variability for that particular year. The figures are prepared in R-4.0.5 (64 bit) windows version and then organized in the MS Office Power point 2016 [Software].

References

1. Dunn, J. C. Well-separated clusters and optimal fuzzy partitions. *Journal of cybernetics* **4**, 95–104 (1974).
2. Bezdek, J. C. A convergence theorem for the fuzzy ISODATA clustering algorithms. *IEEE transactions on pattern analysis and machine intelligence* 1–8 (1980).
3. Ross, T. J. *Fuzzy logic with engineering applications*. vol. 2 (Wiley Online Library, 2004).
4. Rao, A. R. & Srinivas, V. V. Regionalization of watersheds by hybrid-cluster analysis. *Journal of Hydrology* **318**, 37–56 (2006).
5. Pal, N. R. & Bezdek, J. C. On cluster validity for the fuzzy c-means model. *IEEE Transactions on Fuzzy systems* **3**, 370–379 (1995).
6. Ren, M. *et al.* A comparison of flood control standards for reservoir engineering for different countries. *Water* **9**, 152 (2017).
7. Reddy, N. N. *et al.* Legacy data-based national-scale digital mapping of key soil properties in India. *Geoderma* **381**, 114684 (2021).
8. WMO (World Meteorological Organization). *Manual on low-flow estimation and prediction*. (World meteorological organization, 2008). Technical Report no. WMO-No. 1029.
